# Probing Anisotropic Quasiparticle Dynamics and Topological Phase Transitions in Quasi‐1D Topological Insulator ZrTe_5_

**DOI:** 10.1002/advs.202504798

**Published:** 2025-06-20

**Authors:** Yueying Hou, Linze Li, Sutao Sun, Gan Liu, Hongyuan Zhao, Zhiheng Chen, Xuejun Yan, Yang‐Yang Lv, Yurong Yang, Shu‐Hua Yao, Jian Zhou, Y.B. Chen, Ming‐Hui Lu, Vitalyi Gusev, Yan‐Feng Chen

**Affiliations:** ^1^ National Laboratory of Solid State Microstructures Nanjing University Nanjing 210093 China; ^2^ Department of Materials Science and Engineering, College of Engineering and Applied Sciences Nanjing University Nanjing 210093 China; ^3^ Collaborative Innovation Center of Advanced Microstructures Nanjing University Nanjing 210093 China; ^4^ Jiangsu Key Laboratory of Artificial Functional Materials Nanjing University Nanjing Jiangsu 210093 China; ^5^ Department of Physics Nanjing University Nanjing 210093 China; ^6^ Laboratoire d'Acoustique de l'Universite du Mans (LAUM), UMR 6613, Institut d'Acoustigue ‐ Graduate School (A‐GS).CNRS Le Mans Université Le Mans France

**Keywords:** anisotropic polarizaion dependence, quasipartical dynamics, topological insulator, topological phase transition, zirconium pentelluride

## Abstract

The transition metal pentatelluride ZrTe_5_ exhibits rich lattice‐sensitive topological electronic states, and demonstrates great potential in photoelectric and thermoelectric devices. However, a comprehensive investigation of electron‐phonon coupling and phonon scattering process remains limited, despite their importance for transport properties and device optimization. Here, the hot carrier dynamics and a 1.15 THz *A*_g_ mode coherent phonon in ZrTe_5_ are investigated by femtosecond transient spectroscopy across 10–300 K. Notably, polarization‐dependent measurements explicitly decouple a strong anisotropic transient response, which is attributed to the effects of excited‐state electron relaxation and reflectivity modulation by displacive excited coherent phonons. The temperature dependence of electron relaxation time in ZrTe_5_ shows an inflection point, first offering the ultrafast dynamical signature of a temperature‐driven Lifshitz transition. At low temperatures, a long‐lived electron relaxation component emerges in the transient response, providing possible evidence of topological surface states in ZrTe_5_. In addition, the temperature‐dependent coherent phonon is also analyzed, revealing that its scattering is dominated by three‐phonon interactions and exhibits a relatively long lifetime compared to other modes. This work deepens the understanding of ultrafast processes in ZrTe_5_, resolves longstanding questions, paves the way for studying electronic phase transitions, and advances ZrTe_5_'s application in optoelectronic and quantum devices.

## Graphical abstract

This study reveals quasiparticle dynamics in ZrTe_5_. Polarization‐dependent measurements show transient reflectivity anisotropy arises from electron relaxation and lattice vibrations. The first dynamical evidence of a temperature‐induced Lifshitz transition is reported. A long‐lived relaxation at low temperature suggests surface state relaxation. Coherent phonon anharmonicity and scattering mechanisms are identified, advancing the control of ZrTe_5_'s electronic and thermal properties.

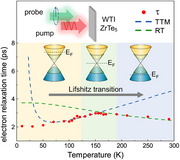

## 1 Introduction

The ultrafast optical pump‐probe technique has emerged as a powerful tool for studying phase transitions in condensed matter systems, where the electronic structure near the Fermi surface plays a crucial role in their equilibrium and quasi‐equilibrium properties.^[^ ^1^, ^2^ ^]^ This technique enables real‐time monitoring of quasiparticle relaxation processes and scattering events, which play a crucial role in the temporal evolution of the Fermi surface.^[^ ^3^, ^4^ ^]^ The complex topology of the Fermi surface is directly related to the novel phenomena observed in transport and optical experiments.^[^ ^5^, ^6^ ^]^ By studying the interactions between non‐equilibrium fermionic quasiparticles(electrons and holes) and bosonic excitations(such as phonons, magnetons, and other low‐energy collective modes), we can gain deeper insights into the microscopic mechanisms involved in phase transitions. These include superconducting transitions,^[^ ^7^, ^8^ ^]^ topological phase transitions,^[^ ^9^ ^]^ and ferromagnetic transitions,^[^ ^10^ ^]^ providing both theoretical foundation and experimental guidance for developing innovative materials and devices.

The transition metal pentatelluride ZrTe_5_ has been studied as a topological insulator and exhibits various topological phase transitions driven by temperature, strain, and coherent phonons.^[^ ^11^, ^12^, ^13^, ^14^, ^15^, ^16^ ^]^ Among the distinctive transport properties of ZrTe_5_, a significant resistivity peak at a temperature *T*_*p*_ ≈ 140 K was first detected and attracted great attention. Later, it was reported that a change in the signs of the Hall and Seebeck coefficients around *T*_*p*_ coincides with the resistivity peak.^[^ ^17^ ^]^ Up to now, the physical mechanism of such resistivity anomaly has still been a matter of debate and remains controversial. Various explanations have been proposed, including the formation of charge density wave,^[^ ^18^ ^]^ polaron models,^[^ ^19^, ^20^ ^]^ semimetal‐semiconductor transitions,^[^ ^21^ ^]^ Lifshitz transitions,^[^ ^16^, ^22^ ^]^ topological phase transitions,^[^ ^23^, ^24^ ^]^ and multiband transport.^[^ ^25^ ^]^ To better understand the anomalies in transport properties and topological phase transitions, it is essential to study the behavior of electrons, phonons, and their coupling within the material. However, although the aforementioned mechanisms all involve changes in quasiparticle dynamics, there has yet to be a study examining the transport anomaly from a dynamical perspective.

To fullfill this gap, we employ the laser‐based ultrafast pump‐probe technique to investigate the hot carrier and phonon dynamics in ZrTe_5_. Our results reveal the quasiparticle dynamics carried by the transient reflectivity signals Δ*R*(*t*)/*R* both experimentally and theoretically: the relaxation of excited state electron through electron–phonon coupling, and the dephasing of $A_{g}^{\text{(1)}}$ mode coherent phonon. Both processes exhibit anisotropic effects on Δ*R*(*t*)/*R*, but with opposite characteristics due to their generation mechanisms. The temperature‐dependent relaxation time of the excited state electrons exhibits an inflection point, which arises from distinct electron‐phonon coupling mechanisms before and after Lifshitz transition. At low temperatures, a long‐lived component of electron relaxation time indicates possible evidence of topological surface states in ZrTe_5_. Additionally, the anharmonicity of the $A_{g}^{\text{(1)}}$ phonon can be well described by a model including lattice thermal expansion, three‐phonon process, and phonon‐electron scattering. Our results clearly investigate the distinct dynamics of different quasiparticles in ZrTe_5_, which not only helps to understand its anomalous transport properties and potential in optoelectronic applications, but also paves a way for studying electronic structural phase transitions in topological materials.

## 2 Results and Discussion

### 2.1 Establishment of A Photoexcitation Dynamics Model

**Figure 1:**
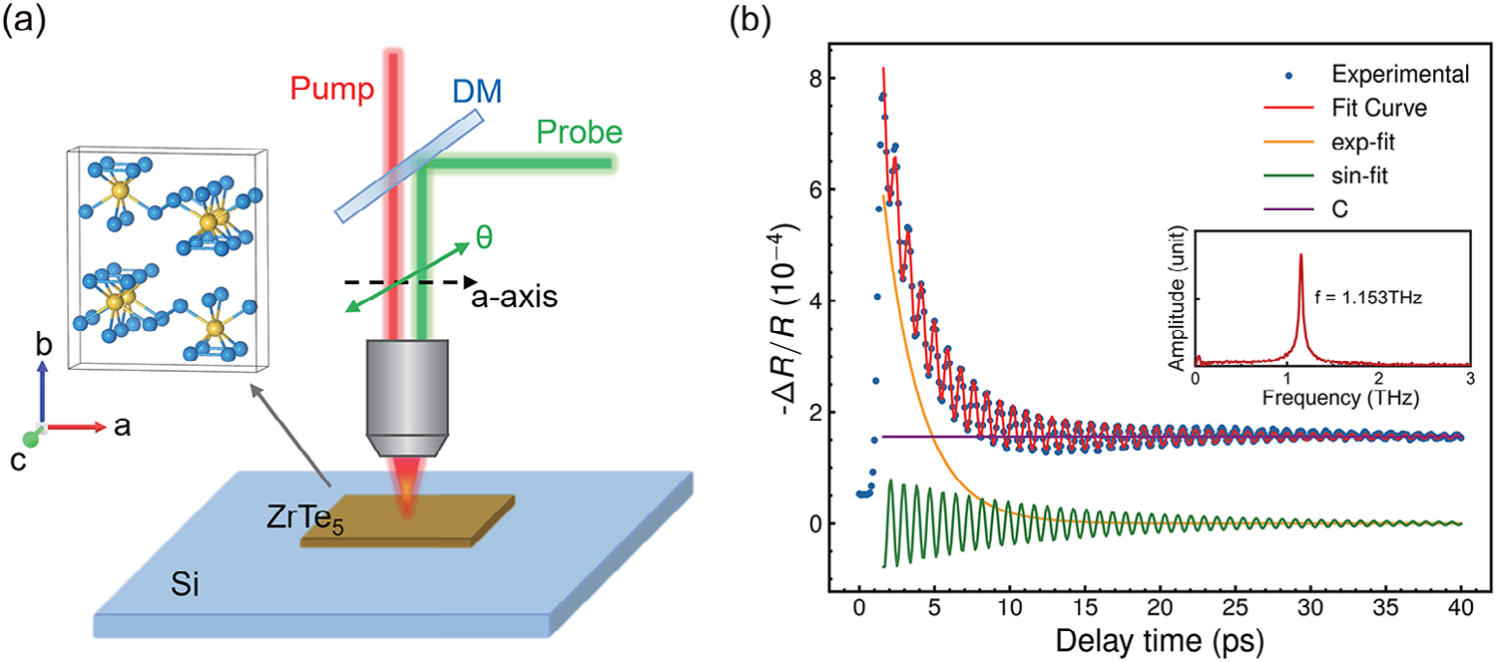
Time‐resolved reflectivity changes of ZrTe_5_. a) Schematic of ultrafast time‐resolved optical reflectivity. A dichroic mirror (DM) was used to combine the pump and probe beam. θ represents the angle between probe polarization and the crystal's c‐axis. b) Representative Δ*R*(*t*)/*R* signal and corresponding fitting for ZrTe_5_ at room temperature. Both pump and probe polarization are parallel to the c‐axis. Inset: The FFT results of this Δ*R*(*t*)/*R* signal.

In this work, a femtosecond pump‐probe system based on Ti: sapphire laser was utilized to get the Δ*R*(*t*)/*R* signal of the sample. Both the pump and probe beam were focused onto the a‐c plane of a cleaved sample surface as shown in **Figure** **1**a. A detailed description of the experimental system can be found in the Experimental Section and Supporting Information. To study the physical properties of materials through ultrafast dynamics, we would like to first analyze the mechanisms behind the generation of transient reflectivity change under our experimental conditions and the information they reveal. Figure 1b depicts a typical Δ*R*(*t*)/*R* signal of ZrTe_5_ measured at room temperature with a pump fluence of 33 µJcm^−2^. It can be observed that the signal exhibits two distinct components: an oscillatory term and an exponentially decaying background. The Fast Fourier Transform (FFT) results shown in the inserted figure reveal a single frequency of approximately 1.15 THz, which corresponds to the frequency of the $A_{g}^{\text{(1)}}$ optical phonon observed in Raman spectroscopy for ZrTe_5_ as shown later in **Figure** **3** The Δ*R*/*R* signal was fitted using the following equation:

(1) $$\begin{array}{cc} \frac{\Delta R(t)}{R} & =\left(A_{e}\exp\left(-\frac{t-t_{0}}{\tau_{e}}\right)+A_{\text{op}}\exp\left(-\frac{t-t_{0}}{\tau_{\text{op}}}\right)\right.\\ & \;\times \left.\sin(\omega t+\phi )+C\right)⊗G\left(t\right) \end{array}$$

here, *A*_e_ and τ_e_ represent the amplitude and relaxation time of the electron relaxation term, *A*_op_, τ_op_, ω, and ϕ denote the amplitude, relaxation time, frequency and initial phase of the coherent optical phonon, respectively. Fitting constant *C* accounts for long‐term relaxation processes such as thermal conduction and electron‐hole recombination. *G*(*t*) is a Gaussian function representing as the pump‐probe cross correlation. In the following subsections, we discuss the photo‐induced coherent phonons and electron dynamics separately, from which the mechanism of photo‐electron‐phonon transition process can be discovered.

**Figure 2:**
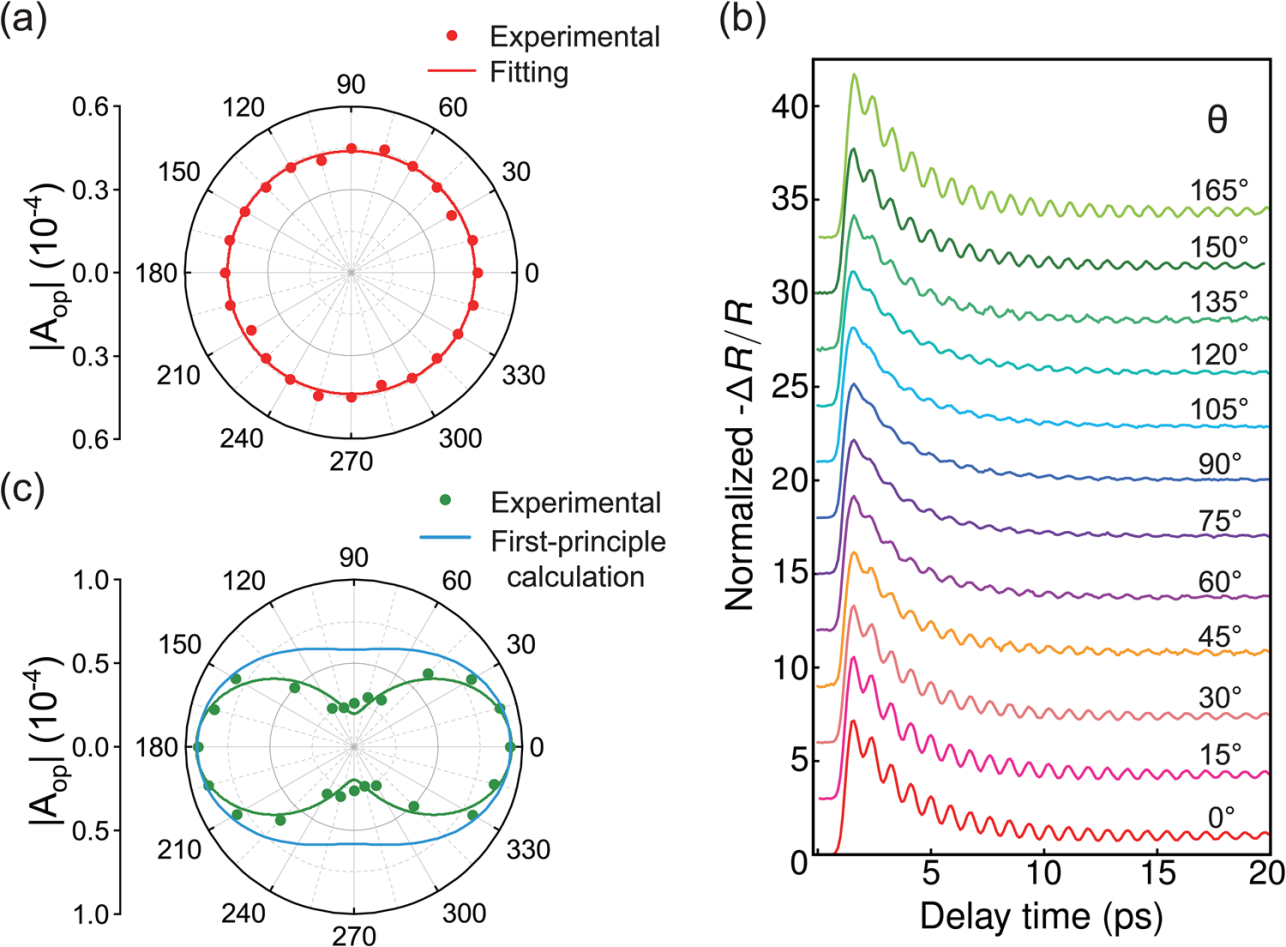
Polarization dependence of transient coherent phonon dynamics. a) The dependence of *A*_op_ on pump polarization with 0° probe polarization. The fitting equation is |*A*_op_| = *B*_1_sin ^2^(θ) + *B*_2_cos ^2^(θ). b) Probe‐polarization‐dependent Δ*R*/*R* signals. θ is the angle between probe polarization and crystal's c‐axis. The result is symmetric about θ = 90°. c) The dependence of fitting parameter *A*_op_ on probe polarization with 0° pump polarization. The blue curve results from first‐principle calculation on $\frac{\partial \varepsilon }{\partial Q}$.

**Figure 3:**
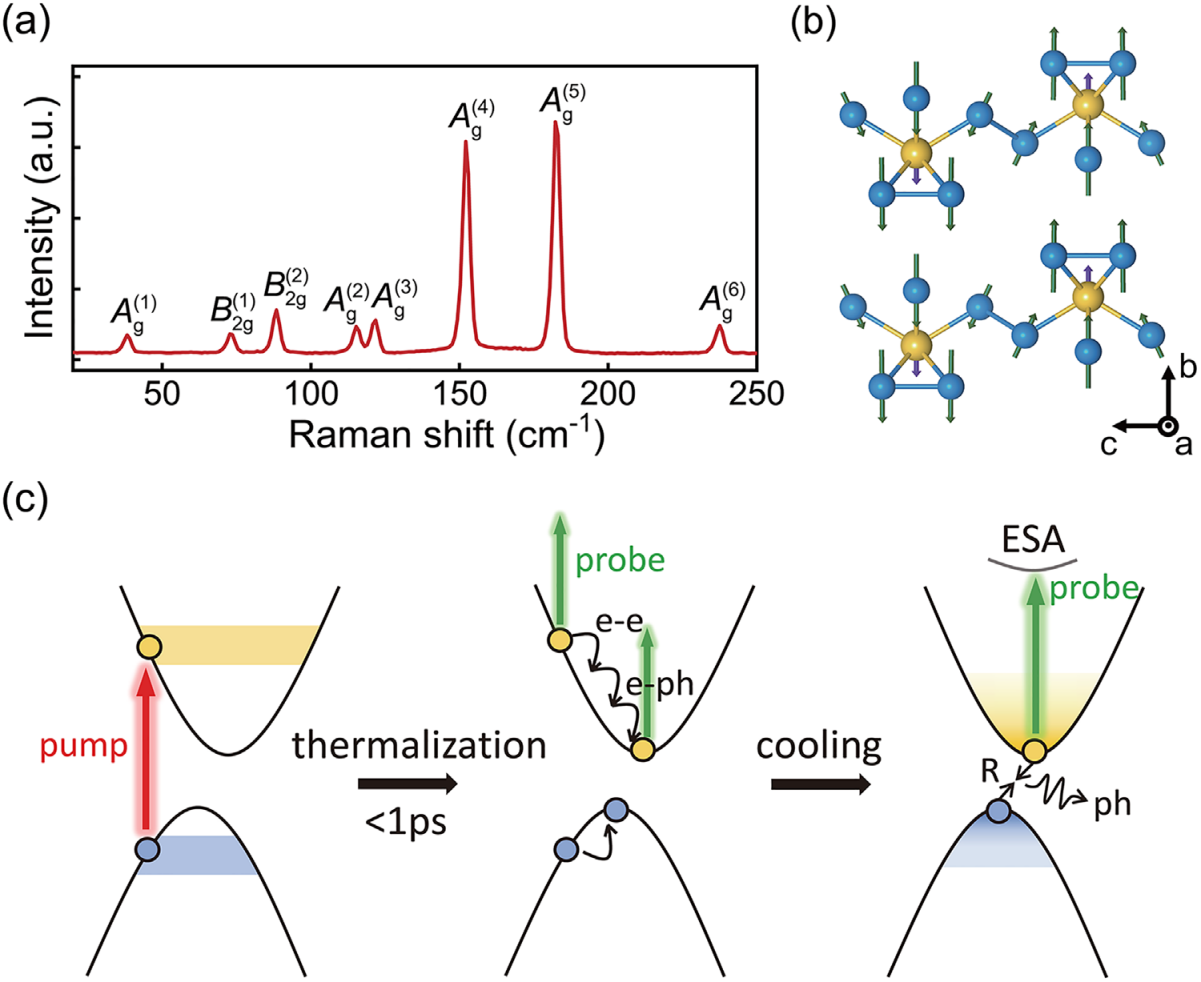
Schematic diagrams of $A_{g}^{\text{(1)}}$ phonon mode and photo‐excited ultrafast dynamics in ZrTe_5_. a) Raman spectra of ZrTe_5_ at room temperature. b) $A_{g}^{\text{(1)}}$ phonon mode of ZrTe_5_ projected onto the b‐c plane with vectors showing the direction and magnitude of the atomic displacements. The coordinate system here represents the abc axes orientations of ZrTe_5_ single crystal. c) Schematic of photoexcitation and phonon‐assisted relaxation process when Fermi energy locates in the valence band of ZrTe_5_. The intensity of color in the yellow and blue regions corresponds to the density of electrons and holes resulting from photoexcitation. Thermalization process corresponds to the rising edge of the signal, while the cooling process corresponds to the decay of the signal.

#### 2.1.1 Photo‐Induced Coherent Phonons

Unlike the spontaneous Raman scattering mechanism in conventional Raman tests, coherent phonons in opaque materials are primarily generated through either Displacive Excitation of Coherent Phonons (DECP) or Impulsive Stimulated Raman Scattering (ISRS), distinguished by whether electronic excitation is real or virtual.^[^ ^26^ ^]^ Previous studies on higher‐frequency *A*_g_ mode in ZrTe_5_ identified DECP as the primary mechanism for coherent phonon generation but did not provide detailed experimental evidence.^[^ ^27^ ^]^ To address this, we conducted experiments by varying the pump laser polarization to confirm that DECP is the dominant mechanism.

Considering a particular phonon mode in ZrTe_5_ single crystal as a damped harmonic oscillator, the main distinction between ISRS and DECP lies in the external force term *F*(*t*):

(2) $$Q^{\prime \prime }\left(t\right)+2\gamma Q^{\prime }\left(t\right)+\omega^{2}Q\left(t\right)=F\left(t\right)$$

here, γ represents the damping constant for the mode, and *Q*(*t*) is the atomic coordinate of $A_{g}^{\text{(1)}}$ mode.

For ISRS,

(3) $$F=\sum_{kl}\left(\frac{\partial \chi }{\partial Q}\;E_{k}E_{l}\right)/2$$

where $\frac{\partial \chi }{\partial Q}$ is related to Raman tensor, and

(4) $$E_{L}=E_{0}e^{-{\left(t-zn/c\right)}^{2}/2\tau_{L}^{2}}\cos\left[\omega_{L}\left(t-zn/c\right)\right]$$

the electric field of pump laser acts as an impulsive force, requiring laser pulse duration τ_*L*_ is much shorter than the oscillation period $\tau_{L}\ll \tau =\frac{2\pi }{\omega }$.^[^ ^28^, ^29^ ^]^ For *A*_g_ modes in ZrTe_5_, Raman tensor $R_{A_{g}}$ has a≠c as shown in Supporting Information.^[^ ^30^ ^]^ Therefore, with the pump light electric field within ac‐plane, the amplitude of coherent phonons (oscillatory term) *A*_op_ should vary with the pump polarization. However, as depicted in **Figure** **2**a, the pump polarization has negligible impact on *A*_op_, indicating that ISRS does not play a major role.

For DECP, $F\left(t\right)=\omega_{0}^{2}Q_{0}\left(t\right)$, where *Q*_0_(*t*) = κ*n*(*t*), κ is the linear coefficient, and *n*(*t*) is the concentration of photo‐excited electrons per unit volume in excited bands. According to the theoretical model,^[^ ^31^ ^]^ *n*(*t*) exhibits a step‐like rise and decays exponentially over time, which can be written as

(5) $$n\left(t\right)=\rho \varepsilon_{\mathrm{pump}}\int_{0}^{\infty }g\left(t-\tau \right)e^{-\beta \tau }\;d\tau$$

here, *g*(*t*) is a normalized pulse shape function such that $\int_{-\infty }^{\infty }g\left(t\right)dt=1$. ρ and β define the rates of *n*(*t*) changes at the surface of the sample, as described by the equation $\dot{n}\left(t\right)=\rho P\left(t\right)-\beta n\left(t\right)$. The first term on the right‐hand side is the rate of generation of excited carriers, which is proportional to the power density, *P*(*t*). The second term is the rate of transfer of electrons back to the ground state. With $\tau_{L}\ll \tau =\frac{2\pi }{\omega }$, electrons are excited in a very short time and dispersed in the momentum space, depending on the pump polarization. Subsequently, the photoexcited electrons relax through electron‐electron scattering, making *n*(*t*) spatially isotropic within 10–100 fs. This time scale agrees with the rising time of experimental results, eventually reaching a quasi‐Fermi distribution. Hence, the $A_{g}^{\text{(1)}}$ phonon mode observed in our experiment is generated via the DECP mechanism. In 2D materials, the collective movement of atoms will strongly modulate the electronic structure, giving rise to periodic signals in Δ*R*/*R* at the phonon mode frequency.

To confirm this conclusion, we conducted additional experiments with the pump polarization aligned parallel to the c‐axis of ZrTe_5_ while rotating the probe polarization. The results, presented in Figure 2b, reveal that the amplitude *A*_op_ varies significantly with the probe polarization angle θ (the angle between the probe polarization and c‐axis), creating an eight‐shaped pattern as shown in Figure 2c. As the external force in the DECP mechanism—due to thermalized electrons—is isotropic, this significant anisotropy arises from the modulation of electronic band structure by coherent phonons, which in turn affects the material's optical properties. Under the assumption that *n*(*t*) in Equation (5) is the dominant driving source of the oscillation, the solution of Equation (2) is

(6) $$\begin{array}{ccc} Q\left(t\right) & = & \frac{{\omega_{0}}^{2}\kappa \rho \varepsilon_{\mathrm{pump}}}{\left(\omega_{0}^{2}+\beta^{2}-2\gamma \beta \right)}\int_{0}^{\infty }g\left(t-\tau \right)\\ & & \times \left[e^{-\beta \tau }-e^{-\gamma \tau }\left(\cos\left(\Omega \tau \right)-\frac{\beta^{\prime }}{\Omega }\sin\left(\Omega \tau \right)\right)\right]d\tau \end{array}$$

here the frequency Ω is given by $\Omega \equiv \sqrt{\omega_{0}^{2}-\gamma^{2}}$ and β′ = β − γ.

In the ultrafast pump‐probe experiments, the transient reflectivity change can be written as

(7) $$\frac{\Delta R\left(t\right)}{R}=\frac{1}{R}\left[\left(\frac{\partial R}{\partial n}\right)n\left(t\right)+\left(\frac{\partial R}{\partial T_{e}}\right)\Delta T_{e}\left(t\right)+\left(\frac{\partial R}{\partial Q}\right)Q\left(t\right)\right]$$

Here, the first term on the right‐hand side is due to *n*(*t*), the excited band carrier density, defined by Equation (5); the second term is due to a change in electron temperature; and the third term is due to the change in the $A_{g}^{\text{(1)}}$ nuclear coordinates, Equation (6). The derivation of Equation (6) is based on the assumption that *n*(*t*) is the dominant source driving *Q*(*t*), but a very similar formal result is obtained if *Q*(*t*) is driven by the temperature change Δ*T*_*e*_(*t*).^[^ ^31^ ^]^

For a probe pulse with the width comparable to that of the pump, *R*(*t*) is actually the convolution of *Q*(*t*) with the probe pulse, and the measurement is an average over the pulse. Therefore, the fractional change in reflectivity can be written as:

(8) $$\begin{array}{cc} \frac{\Delta \bar{R}\left(t\right)}{R}\;\;=\;\; & A\int_{0}^{\infty }G\left(t-\tau \right)e^{-\beta \tau }\;d\tau \\ & +B\frac{\omega_{0}^{2}}{\omega_{0}^{2}+\beta^{2}-2\gamma \beta }\int_{0}^{\infty }G\left(t-\tau \right)\\ & \times \left[e^{-\beta \tau }-e^{-\gamma \tau }\left(\cos\left(\Omega \tau \right)-\frac{\beta^{\prime }}{\Omega }\sin\left(\Omega \tau \right)\right)\right]\;d\tau \\ & +C\int_{0}^{\infty }G\left(t-\tau \right)\;d\tau \end{array}$$

where *G*(*t*) is the convolution of the pump and probe pulse. The last term represents long‐lived processes whose relaxation times exceed our measurement time. *A* and *C* represent the amplitude of exponentially decaying term and long‐lived term, corresponding to the first term on the right‐hand side of Equation (7). *B* represents the amplitude of the oscillatory term, corresponding to the third term on the right‐hand side of Equation (7). By comparing with Equation (7) and Equation (6):

(9) $$\begin{array}{cc} B & =\frac{1}{R}\left(\frac{\partial R}{\partial Q}\right)\kappa \rho E_{pump}\\ & =\frac{1}{R}\left[\left(\frac{\partial R}{\partial \varepsilon_{1}}\right)\left(\frac{\partial \varepsilon_{1}}{\partial Q}\right)+\left(\frac{\partial R}{\partial \varepsilon_{2}}\right)\left(\frac{\partial \varepsilon_{2}}{\partial Q}\right)\right]\kappa \rho E_{pump} \end{array}$$

Thus, the ratio of the oscillation term amplitudes under different probe polarization is:

(10) $$\frac{B_{x}}{B_{y}}=\frac{R_{y}\left(\frac{\partial R_{x}}{\partial Q}\right)}{R_{x}\left(\frac{\partial R_{y}}{\partial Q}\right)}=\frac{R_{y}\left[\left(\frac{\partial R_{x}}{\partial \varepsilon_{1x}}\right)\left(\frac{\partial \varepsilon_{1x}}{\partial Q}\right)+\left(\frac{\partial R_{x}}{\partial \varepsilon_{2x}}\right)\left(\frac{\partial \varepsilon_{2x}}{\partial Q}\right)\right]}{R_{x}\left[\left(\frac{\partial R_{y}}{\partial \varepsilon_{1y}}\right)\left(\frac{\partial \varepsilon_{1y}}{\partial Q}\right)+\left(\frac{\partial R_{y}}{\partial \varepsilon_{2y}}\right)\left(\frac{\partial \varepsilon_{2y}}{\partial Q}\right)\right]}$$

To further validate this model, we first performed first‐principles calculations to determine the partial derivative of the dielectric constant ε with respect to atomic displacement *Q* for the $A_{g}^{\text{(1)}}$ mode at 1.9 eV (schematic of $A_{g}^{\text{(1)}}$ optical phonon mode was shown in Figure 3b). The result, applied to Equation (10) was shown as the blue curve in Figure 2c. This curve is generally consistent with the experimental data and aligns well with the fitting results when analyzed from an anisotropic perspective. Small deviations may stem from differences in lattice parameters between the experimental and calculated values and the limitations of theoretical models in van der Waals materials. Additionally, polarization‐dependent Raman spectroscopy confirmed the reliability of the first‐principles calculations. Subsequently, we implemented real‐time time‐independent density functional theory (rt‐TDDFT) simulations, investigating the displacive excitation amplitudes of *A*_g_ modes by a femtosecond laser pulse with DECP mechanism. More details are shown in the Supporting Information.

#### 2.1.2 Photoexcited Electrons

**Figure 4:**
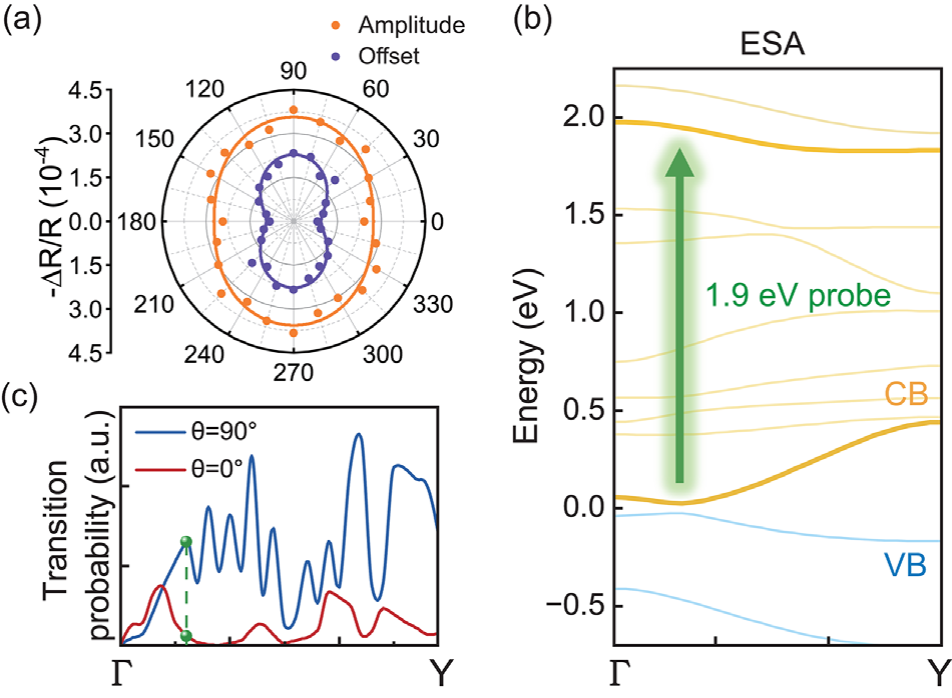
Theoretical and experimental confirmation of polarization dependence on the transient reflectivity change caused by excited state electrons. a) The dependence of fitting parameters Amplitude *A*_*e*_ and Offset *C* on probe polarization with 0° pump polarization. b) Electronic band structure of ZrTe_5_ near the Γ point in the momentum space. The relaxed pump‐generated electrons in the lowest conduction band (the lower thick yellow line) were excited by probe photons to the high energy band (the upper thick yellow line) that is ≈ 1.9 eV above the CBM. This process corresponds to ESA. c) Light‐polarization‐resloved transition probability for ESA transition shown in (b). The green dots represent the wave vector of the CBM.

After demonstrating that the source of the oscillation term is caused by coherent phonons and discussing its anisotropy in relation to Raman scattering, we now turn to the exponential decay term in the Δ*R*(*t*)/*R* signal. In probe polarization‐dependent experiments, Δ*R*/*R* remains negative under all conditions, indicating a photo‐induced absorption (PA) mechanism. Since the probe wavelength is shorter than the pump (more details on the optics could be found in Experimental Section), bandgap renormalization and free‐carrier absorption are not responsible of PA, suggesting that excited state absorption (ESA) is the main cause.^[^ ^32^ ^]^ The variation of *A*_e_ with θ is depicted in **Figure** **4**a. In contrast to *A*_op_, *A*_e_ reaches a maximum when θ = 90°. According to the ESA mechanism, photoexcited electrons in the conduction band absorb probe photons for transition to higher energy bands.^[^ ^33^, ^34^ ^]^ At room temperature, the Fermi energy lies within the valence band of ZrTe_5_, resulting in a significantly altered density of conduction band electrons while leaving the hole density nearly unchanged. Thus, we only consider the ESA of the electrons in the conduction band. Theoretical calculations of polarization‐dependent transition probability from the conduction band minimum (CBM) to higher‐energy bands near 1.9 eV at the Γ‐point are shown in Figure 4c. It confirms that the probability is highest when E ∥ a (θ = 90°) and lowest when E ∥ c (θ = 0°), indicating that more a‐polarized light would be absorbed per unit volume compared to c‐polarized light. In narrow bandgap bulk materials, light transmission can be neglected due to strong absorption. Therefore, take the energy conservation law into consideration, Δ*A* = −Δ*R*. The greater the change in the surface absorption, the correspondingly larger the change in reflectivity. Dividing the different polarized light into two orthogonal components along the a‐axis and c‐axis, we could express the absorption change by |Δ*R*| = |Δ*A*| = Δ*A*_*a*_sin ^2^(θ) + Δ*A*_*c*_cos ^2^(θ). Δ*R* reaches its maximum when E ∥ a, which is consistent with our experimental observations. Notably, the fitting constant *C* exhibits even larger in‐plane anisotropy than *A*_e_, suggesting that the photoexcited electronic structure becomes increasingly anisotropic following the relaxation. Based on these findings, we propose a comprehensive model of photoexcited dynamics in ZrTe_5_ and other narrow‐bandgap semiconductors. A schematic representation is shown in Figure 3c.

### 2.2 Temperature‐dependent Lifshitz Transition in ZrTe_5_

Since the meaning of each term in transient reflectivity signal is clear, we get the measurement data of Δ*R*(*t*)/*R* at different temperatures (10–300 K), and fit the experimental results with Equation (1). As temperature decreases, the decay of signal background gradually deviates from the single‐exponential trend, and a long‐lived decay process becomes increasingly significant (The fitting results with single‐exponential background for the entire temperature range can be found in Supporting Information). It indicates that more electron‐phonon interaction processes should be considered. Therefore, we applied a bi‐exponential decay model (Equation (11)) to this dataset, achieving good agreements across the entire temperature spectrum:

(11) $$\begin{array}{cc} \frac{\Delta R(t)}{R} & =\left(A_{\text{ep}}\exp\left(-\frac{t-t_{0}}{\tau_{\text{ep}}}\right)+A_{\text{e2}}\exp\left(-\frac{t-t_{0}}{\tau_{\text{e2}}}\right)\right.\\ & \;\left.+A_{\text{op}}\exp\left(-\frac{t-t_{0}}{\tau_{\text{op}}}\right)\sin\left(\omega t+\phi \right)+C\right)⊗G\left(t\right). \end{array}$$

Here we define τ_ep_ is shorter than τ_e2_.

**Figure 5:**
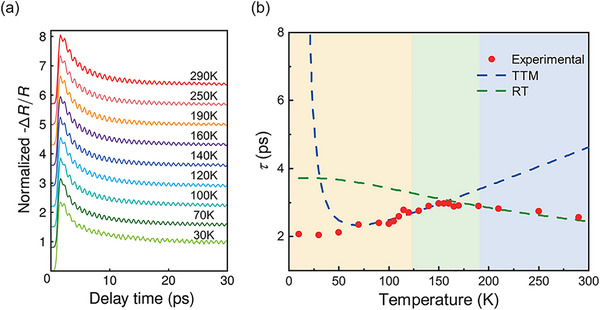
Temperature dependence of transient reflectivity change. a) Part of the temperature‐dependent signal. Full data and fitting process can be found in Supporting Information. b) The temperature dependence of the short‐lived decay time in ZrTe_5_ (red dots) compared to the modified TTM (blue dashed line) and RT simulation (green dashed line). The colored background represents the Fermi level positioned at different energy bands, according to earlier studies.

The relaxation time of the short‐lived process (written as τ_ep_) as a function of temperature is illustrated in **Figure** **5**b. An inflection point is observed around 150 K, which is close to the temperature at which the Lifshitz transition occurs in ZrTe_5_.^[^ ^16^ ^]^ Thus, we speculated that the emergence of an inflection point is related to the movement of the Fermi level. At relatively low temperatures, the Fermi level lies in the conduction band of ZrTe_5_. Thus, excited state electrons relax within conduction bands, and we fitted this process with the Two Temperature Model (TTM). On the contrary, at temperatures above the transition temperature, the Fermi level lies in the valance band. Excited state electrons have to relax through the band gap assisted by high‐frequency phonons. So the Rothwarf‐Taylor (RT) model was used in this condition. Both are related to electron–phonon coupling, the main difference between these two models lies in the fact that in RT model, the relaxation of electrons depends on the high‐frequency phonons of band gap energy. So their electronic relaxation exhibits different characteristics under the conditions of varying temperature and pump laser fluence.

For temperatures below 105 K, the Fermi level of ZrTe_5_ lies within the conduction band. Excited electrons relax to energies near the Fermi level by transferring energy to phonons, consistent with the TTM. The instantaneous energy relaxation time is defined by^[^ ^35^ ^]^:

(12) $$\tau_{\text{ep}}\left(T_{e},T_{l}\right)\equiv \frac{U_{e}\left(\infty \right)-U_{e}}{\left(dU_{e}/dt\right)}=\frac{\gamma (T_{e}^{2}-T_{l}^{2})}{2g\left(T_{l}\right)\left(T_{e}-T_{l}\right)}$$

where $U_{e}\left(\infty \right)\approx \frac{1}{2}\gamma T_{l}^{2}$, $\frac{dU_{e}}{dt}=H\left(T_{e},T_{l}\right)=f\left(T_{e}\right)-f\left(T_{l}\right)$.

Under low pump fluence, *T*_*e*_ − *T*_*l*_ ≪ *T*_*l*_, we have:

(13) $$\tau_{\text{ep}}=\frac{\gamma (T_{e}+T_{l})}{2g(T_{l})}≅\frac{\gamma (T_{l})}{g(T_{l})}$$

And the electron‐phonon coupling function is defined as *g*_*ep*_(*T*) ≡ *df*/*dT*, with

(14) $$f\left(T\right)=4g_{\infty }{\left(\frac{T}{\Theta_{D}}\right)}^{5}\int_{0}^{\Theta_{D}/T}\frac{x^{4}}{e^{x}-1}\;dx$$

here, Θ_*D*_ is the Debye temperature, γ is given by *C*_e_ = γ*T*, with $\gamma =\frac{\pi^{2}}{3}k_{B}^{2}N\left(E_{F}\right)\approx 1.18\;mJ\cdot mol^{-1}\cdot K^{-2}$ (A later modification was shown in Supporting Information). *N*(*E*_*F*_) was calculated by Gao et al.^[^ ^36^ ^]^

Consequently, the fitted temperature‐dependent relaxation time τ_ep_ is shown as a blue dashed line in Figure 5b. Fitting parameter *g*_∞_ ≈ 6 × 10^16^ *W* · *m* ^−3^ · *K* ^−1^ stands for the electron‐phonon coupling constant. The discrepancy between experimental results and TTM simulation at low temperatures emerges because TTM neglects e–e thermalization processes, which is quite general for simple metals.^[^ ^37^ ^]^ Further discussion of the limitations of the TTM at low temperatures and its applicable temperature range can be found in the Supporting Information.

For temperatures above 195 K, the Fermi level of ZrTe_5_ moves into the valence band. In this regime, the dynamics of photoexcited electron relaxation are influenced by the presence of a narrow gap in the density of states. According to the RT model, excited electrons recombine with holes by creating high frequency phonons, which in turn re‐excite electron hole pairs. Thus we have quasi‐stationary distributions of quasiparticles *n*_*s*_ satisfying:

(15) $$\begin{array}{cc} & Rn_{s}^{2}=\eta N_{s}\\ & n_{s}=\frac{R}{4\eta }\left(\sqrt{1+\frac{16R}{\eta }n_{0}+\frac{8R}{\eta }N_{0}-1}\right) \end{array}$$

where *n*_0_ and *N*_0_ represent the initial concentrations of electron‐hole pairs and high frequency phonons which increase linearly with pump fluence *F*.^[^ ^38^ ^]^ And *N*_*s*_ is the quasi‐stationary distributions of high‐frequency phonons.

The initial relaxation rate $\tau_{ep}^{-1}$ is given in the RT model by

(16) $$\tau_{ep}^{-1}=\frac{2R\gamma \left(n_{s}+n_{T}\right)}{\eta^{2}\left(1+2\gamma /\eta \right)}$$

where, *n*_*T*_ denotes the density of thermally equilibrated electron‐hole pairs. In a narrow band insulator, *n*_*T*_ can be written as:

(17) $$n_{T}≃T^{p}\exp\left(-\frac{E_{g}}{2T}\right)$$

where *p* is a parameter of the order of 1, which depends on the exact shape of the density of states near the gap edge. For a constant excitation fluence, τ_ep_ is given by

(18) $$\tau_{ep}^{-1}\left(T\right)=\Gamma \left[\delta {\left(\varepsilon n_{T}+1\right)}^{-1}+2n_{T}\right]$$

here δ and ε are constants dependent only on the photoexcitation fluence.^[^ ^3^ ^]^

Using Equations (17) and (18), we can derive the fitting curve of τ_ep_ with experimental data above 195 K, represented by green dashed line in Figure 5b. A bandgap of ≈ 20 meV was used in the fitting as observed by H. Xiong at al.^[^ ^39^ ^]^ The good agreement with both models and the experimental results indicates a change in electronic dynamics associated with the Lifshitz transition in ZrTe_5_.

Furthermore, it is evident from the expressions for Equations (13) and (16) that τ_ep_ also depends on pump fluence. To further verify it, we conducted pump‐fluence‐dependent experiments. Specifically, the experimental results for T < 105 K indicate that *T*_*e*_ − *T*_*l*_ ≪ *T*_*l*_ is not valid throughout the entire time range, thus requiring a modification of TTM. Detailed discussion can be found in the Supporting Information.

For the relatively long‐lived process, its emergence and amplitude growth at low temperatures(shown in Figure S5, Supporting Information) may indicate significant topological surface state signals at these temperatures. Detailed discussion can be found in the Supporting Information. As a weak topological insulator, the ground state ZrTe_5_ possesses topological surface states only on particular side surfaces. Consequently, incident light on the mechanically exfoliated step edges may provide insights into these topological surface states. However, this inference necessitates more direct experimental evidence, such as: using THz pump‐probe to couple to the surface states selectively; using circularly polarized excitation to exploit spin‐momentum locking; and using Tr‐ARPES to investigate the surface state dynamics.

### 2.3 Phonon Anharmonicity in ZrTe_5_

**Figure 6:**
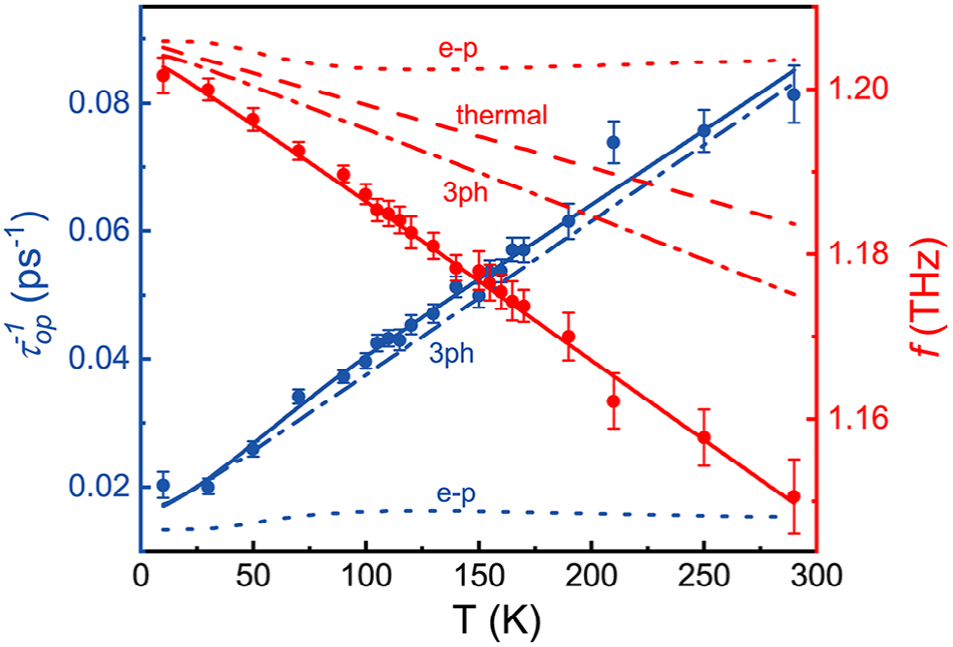
Temperature dependence of vibrational frequency *f* = ω/2π and dephasing rate $\tau_{\text{op}}^{-1}$. Note that the red and blue solid lines are fitted by Equations (19) and (23), respectively. The contributions from pure thermal expansion, three‐phonon process and phonon‐electron scattering are shown by three types of dashed lines separately. The anharmonic coefficients of four‐phonon process (*D*_4ph_ in Equation (21)) are an order of magnitude smaller than those of three‐phonon process, thus not presented in the figure.

In the temperature‐dependent experiments, we also studied the dephasing of $A_{g}^{\text{(1)}}$ mode low‐frequency optical phonons, reflecting the phonon scattering process. It was not previously reported in Raman experiments due to resolution limitations.^[^ ^40^ ^]^ We found that the dephasing time of $A_{g}^{\text{(1)}}$ mode is an order of magnitude larger than that of $A_{g}^{\text{(3)}}$ mode, which is consistent with the result of first‐principles calculations and indicates a greater impact on thermal conduction.^[^ ^27^, ^41^ ^]^ **Figure** **6** shows the vibrational frequency *f* and corresponding dephasing time τ_op_ of the $A_{g}^{\text{(1)}}$ mode as a function of temperature. With phonon‐electron scattering concerned due to low experimental temperature, the temperature‐dependent phonon frequency can be expressed as:

(19) $$\omega \left(T\right)=\omega_{0}+\Delta_{0}\left(T\right)+\Delta \omega_{\mathrm{ph}-\mathrm{ph}}\left(T\right)+\Delta \omega_{\mathrm{ph}-e}\left(T\right)$$

here, ω_0_ is the intrinsic phonon frequency at 0 K, and Δ_0_(*T*) is the frequency shift caused by thermal expansion which can be expressed as:

(20) $$\Delta \omega_{E}\left(T\right)=\omega_{0}\left[\exp\left(-\gamma \int_{0}^{T}\alpha_{V}\left(T^{\prime }\right)\;dT^{\prime }\right)-1\right]$$

where γ is the $\mathrm{Gr}\ddot{u}\mathrm{neisen}$ parameter and α_*V*_ is the volume thermal expansion coefficient. Based on earlier first‐principle calculations, we used an average parameter γ = 1.23 in our fitting.^[^ ^42^ ^]^ For α_*V*_ of ZrTe_5_, we utilized the sum of the linear thermal expansion coefficients along the a, b, and c axes as reported earlier.^[^ ^43^ ^]^

Anharmonic phonon‐phonon decay and phonon‐electron scattering are two main channels of optical phonon decay, as described by Δω_ph‐ph_ and Δω_ph‐e_ in Equation (19), respectively.

Δω_ph‐ph_ can be written as:

(21) $$\begin{array}{cc} \Delta \omega_{\mathrm{ph}-\mathrm{ph}}\left(T\right) & =D_{3\mathrm{ph}}\left[1+\frac{2}{\exp\left(\frac{\hbar \omega_{0}}{2k_{B}T}\right)-1}\right]\\ & \;+D_{4\mathrm{ph}}\left[1+\frac{3}{\exp\left(\frac{\hbar \omega_{0}}{3k_{B}T}\right)-1}+\frac{3}{{\left(\exp\left(\frac{\hbar \omega_{0}}{3k_{B}T}\right)-1\right)}^{2}}\right] \end{array}$$

where the first and second terms represent the three‐phonon and four‐phonon processes, respectively. The numerators 2 (or 3) indicate an optical phonon (ω_0_) decays into two (or three) acoustic phonons with identical frequencies $\frac{\omega_{0}}{2}$ (or $\frac{\omega_{0}}{3}$). *D*_3ph_ and *D*_4ph_ reveal the contributions of these anharmonic processes.^[^ ^44^, ^45^ ^]^ However, the temperature dependence of the four‐phonon coupling term exhibits a parabolic profile, becoming more significant at high temperatures, which was not obvious in our experimental data.

For Δω_ph‐e_, it describes a process in which an optical phonon decays into an electron‐hole pair via phonon‐mediated electron excitation. Thus, by introducing phonon–electron scattering guided by the Fermi–Dirac distribution function, this term can be expressed as:

(22) $$\Delta \omega_{\mathrm{ph}-e}\left(T\right)=D_{\mathrm{phe}}\left[\frac{1}{\exp(\frac{\hbar \omega_{e}}{k_{B}T})+1}-\frac{1}{\exp(\frac{\hbar \omega_{e}+\hbar \omega_{0}}{k_{B}T})+1}\right]$$

where ℏω_*e*_ is the energy of the electron's initial state with respect to the Fermi energy *E*_F_.

Similarly, the temperature‐dependent phonon dephasing rate can be described as:

(23) $$\begin{array}{cc} \Gamma (T) & =\Gamma_{0}+G_{3\mathrm{ph}}\left[1+\frac{2}{\exp\left(\frac{\hbar \omega_{0}}{2k_{B}T}\right)-1}\right]\\ & \;+G_{\mathrm{phe}}\left[\frac{1}{\exp(\frac{\hbar \omega_{e}}{k_{B}T})+1}-\frac{1}{\exp(\frac{\hbar \omega_{e}+\hbar \omega_{0}}{k_{B}T})+1}\right] \end{array}$$

where Γ_0_ is the phonon dephasing rate at 0 K.

As shown in Figure 6, the three‐phonon process predominantly contributes to the temperature‐induced redshift of $A_{g}^{\text{(1)}}$ mode. As a layered 2D material, ZrTe_5_ features weak van der Waals bonding along the b‐axis. Consequently, it exhibits anisotropic bonding nature due to strong in‐plane covalent bonding and weak interlayer interactions, giving rise to soft optical modes coupling with heat‐carrying acoustic phonons, and thereby reducing the lattice thermal conductivity and increasing lattice anharmonicity.^[^ ^46^ ^]^ As the population of thermally excited phonons increases with temperature, the larger dephasing rate is also due to increased three‐phonon scattering.

However, although the $A_{g}^{\text{(1)}}$ mode coherent phonon in ZrTe_5_ is excited through strong electron–phonon coupling, it does not exhibit a correlation with the temperature‐induced electronic phase transition through the temperature dependent τ_op_ and *f*. The fitting results shown in Figure 6 also indicate that phonon–electron scattering does not play an important role in the coherent phonon decay of ZrTe_5_.

## 3 Conclusion

In summary, we investigated the dynamics of hot carriers and coherent phonons in ZrTe_5_ by femtosecond transient optical spectroscopy across various light polarizations and temperatures. The anisotropy in transient reflectivity, attributed to Displacive Excitation of Coherent Phonons and Excited State Absorption, was decoupled by polarization‐dependent experiments. Additional first‐principles calculations confirmed that the anisotropy of coherent phonons originates from the modulation of dielectric constant by atomic displacement, which could also be observed in Raman spectroscopy. This provides theoretical support for employing ultrafast pump‐probe techniques to study time‐resolved Raman dynamics. Based on the above schematic diagram, temperature‐dependent carrier and phonon dynamics across from 10 to 300 K were also studied. Particularly, the temperature dependence of the short‐lived process provides the first evidence of Lifshitz transition in ZrTe_5_ from the perspective of ultrafast dynamics. A long‐lived component that appears at low temperatures is likely associated with the topological surface state. Furthermore, the dephasing process of $A_{g}^{\text{(1)}}$ mode coherent phonon was well‐fitted with a model including lattice thermal expansion, three‐phonon process, and phonon‐electron scattering, and shows no abrupt change due to the electronic transition.

This work comprehensively studied the quasiparticle dynamics of ZrTe_5_, and demonstrated that ultrafast spectroscopy enables the exploration of novel physical states, such as topological transitions from the perspective of quasiparticle dynamics. Specifically, the characterization of ultrafast electron dynamics provides critical insights into the photoelectric effect and facilitates the discovery of new photoelectric materials, while the light‐induced coherent phonon potentially allows transient control of new phases, including transient superconductivity, ferroelectric/ferromagnetic states, and terahertz emission. Furthermore, this mechanism sheds light on phonon dynamics, paving the way for the discovery of advanced thermoelectric materials, thermal management materials, and thermal resistance materials.

## 4 Experimental Section

### Sample Preperation

High quality ZrTe_5_ single crystals were grown by the chemical vapor transport method with iodine (I_2_) as the transport agent.^[^ ^47^ ^]^ First, polycrystalline ZrTe_5_ samples were synthesized by a solid‐state reaction with high pure Zr (GRINM, 5 N) and Te (Alfa Aesar, 5 N) powders in a sealed silica tube (*P* ∼ 4 × 10^−6^Torr) at about 500° C for 7 days. Then the mixture of prepared ZrTe_5_ and *I*_2_ (about 5 mg L^−1^) powders were loaded into the sealed evacuated quartz tube (*P* ≈ 4 × 10^−6^Torr), and put into a two‐zone furnace with a temperature profile of 450–500° C to grow crystals. The millimeter‐sized crystals with metallic‐luster were obtained successfully after growing for over 10 days.

### Ultrafast Pump‐Probe Experiment

In the experimental setup shown in Figure S9 (Supporting Information), the femtosecond laser pulses, centered at 800 nm, were generated by a Ti:sapphire laser operating at 76 MHz repetition rate. The light beam from the laser was split into two parts. The first part was used as a pump sequence of pulses, while the second was directed to an optical parametric oscillator (OPO) to generate a synchronized sequence of probe pulses at 650 nm. The pump beam was modulated by an electro‐optic modulator (EOM) at a frequency of 1 MHz, and then compressed by two pairs of chirped mirrors to reach a pulse duration of 160 fs before the sample. The time delay between the probe and pump beams was controlled by a mechanical delay stage installed in the pump arm. The collinear pump and probe beams were focused onto the sample surface using a 4× objective (N4X‐PF, Nikon), resulting in spot diameters of approximately 10µm. The pulse fluencies of pump and probe were 33 µJ. cm^−^^2^ and 0.4 µJcm^−2^, respectively. The sample was mounted in a Montana cryostat, allowing precise control of temperatures from 10 to 300 K. The modulated signal was amplified using a lock‐in amplifier and subsequently collected by a data acquisition card. To accurately analyze the ultrafast dynamics mechanism, phase correction was performed on the signal by adjusting the absolute value of the phase of the reflected pump signal in the lock‐in amplifier, ensuring that its phase remains constant at zero delay time.

### First‐Principles Calculations

The optical properties of ZrTe_5_ were calculated by the density functional theory in the generalized gradient approximation implemented in the Vienna ab initio simulation package code.^[^ ^48^, ^49^ ^]^ The projected augmented wave method^[^ ^50^, ^51^ ^]^ and the Perdew‐Burke‐Ernzerhof revised for solids (PBEsol) exchange‐correlation^[^ ^52^ ^]^ were used in the calculations. The experimental lattice constants^[^ ^43^ ^]^ of ZrTe_5_ were used in the calculations but the atomic coordinates were optimized with a maximal residual force of 0.0005 eV. Å^−1^. A k‐mesh of 9× 9 ×3 was used in the structural optimization and phonon calculations while a denser k‐mesh of 21 × 21 × 7 was used in the optical calculations. The plane‐wave cutoff energy was 350 eV throughout the calculations. The zone‐centered phonon modes were calculated with the assistance of the Phonopy code.^[^ ^53^ ^]^ The frequency‐dependent optical transition matrix and dielectric functions were calculated in the longitudinal form implemented in the VASP code.^[^ ^54^ ^]^

### Statistical Analysis

The experimental data were analyzed using standard statistical methods to ensure reliability and reproducibility. The results presented in Figures 2b and 5a, obtained at varying polarizations and temperatures, were normalized to their respective peak values for relaxation characteristics comparison. For the vibrational frequency *f* = ω/2π and dephasing rate $\tau_{\text{op}}^{-1}$, the fitting parameters were reported as mean ± SD (standard deviation), as shown in Figure 6. All measurements were repeated twice independently to assess variability across experiments. To enhance the signal‐to‐noise ratio (SNR), each data point was averaged over 600 independent tests, ensuring robust data collection. Notably, this averaging process was performed prior to statistical analysis and is not included in the computation of mean and standard deviation. Statistical computations were conducted using Python and Origin software, and significance levels were set at *p* < 0.05 unless otherwise stated.

## Conflict of Interest

The authors declare no conflict of interest.

## Supporting information

Supporting Information

## Data Availability

The data that support the findings of this study are available from the corresponding author upon reasonable request.
