# Supplementary material for: Probing Anisotropic Quasiparticle Dynamics and Topological Phase Transitions in Quasi‐1D Topological Insulator ZrTe5
Source: Adv Sci (Weinh). 2025 Jun 20;12(31):e04798. doi: 10.1002/advs.202504798 (PMC12376609; doi:10.1002/advs.202504798)
Supplement: Supplementary file 1 — Supporting Information [file ADVS-12-e04798-s001.pdf]

ADVANCED SCIENCE
Open Access

Supporting Information

for *Adv. Sci.*, DOI 10.1002/advs.202504798

Probing Anisotropic Quasiparticle Dynamics and Topological Phase Transitions in Quasi-1D Topological Insulator $ZrTe_5$

Yueying Hou, Linze Li, Sutao Sun, Gan Liu, Hongyuan Zhao, Zhiheng Chen, Xuejun Yan, Yang-Yang Lv*, Yurong Yang, Shu-Hua Yao, Jian Zhou, Y.B. Chen, Ming-Hui Lu, Vitalyi Gusev and Yan-Feng Chen*

Supporting Informations for:

Probing Anisotropic Quasiparticle Dynamics and Topological Phase Transitions in Quasi-1D Topological Insulator $ZrTe_5$

Yueying Hou, Linze Li, Sutao Sun, Gan Liu, Zhiheng Chen, Hongyuan Zhao, Xuejun Yan, Yang-Yang Lv*, Yurong Yang, Shu-Hua Yao, Jian Zhou, Y.B. Chen, Ming-Hui Lu, Vitalyi Gusev, Yan-Feng Chen*

Y. Hou, L. Li, S. Sun, G. Liu, H. Zhao, Z. Chen, X. Yan, Y.-Y. Lv, Y. Yang, S.-H. Yao, J. Zhou, Y. B. Chen, M.-H. Lu, Y.-F. Chen
National Laboratory of Solid State Microstructures, Nanjing University, Nanjing 210093, China
Email Address: xjyan@nju.edu.cn; lvyangws0801@nju.edu.cn

Y. Hou, L. Li, H. Zhao, Z. Chen, X. Yan, Y.-Y. Lv, Y. Yang, S.-H. Yao, J. Zhou, M.-H. Lu, Y.-F. Chen
Department of Materials Science and Engineering, College of Engineering and Applied Sciences, Nanjing University, Nanjing 210093, China

S. Sun, G. Liu, Y. B. Chen
Department of Physics, Nanjing University, Nanjing 210093, China

L. Li, Y. Yang, M.-H. Lu, Y.-F. Chen
Collaborative Innovation Center of Advanced Microstructures, Nanjing University, Nanjing 210093, China

L. Li, Y. Yang, M.-H. Lu
Jiangsu Key Laboratory of Artificial Functional Materials, Nanjing University, Nanjing, Jiangsu 210093, China

V. Gusev
Laboratoire d'Acoustique de l'Universite du Mans (LAUM), UMR 6613, Institut d'Acoustigue - Graduate School (A-GS).CNRS, Le Mans Université, France

In the supplementary materials, we will elaborate the following issues: 1) Experimental and first-principles calculation results of polarization-dependent $A_g^{(1)}$ mode Raman intensity in $ZrTe_5$. 2) Results of real-time time-independent density functional theory (rt-TDDFT) simulations. 3) The necessity of bi-exponential fitting for low temperature range. 4) The limitations of the Two-Temperature Model (TTM) at low temperatures and its applicable temperature range in this work. 5) Analysis of characteristic parameters for the short-lived process dependent on pump-fluence. 6) Discussion of the potential mechanism of the long-lived process. 7) Experimental setup of ultrafast pump-probe system.

1 Experimental and first-principles calculation results of polarization-dependent $A_g^{(1)}$ mode Raman intensity in $ZrTe_5$.

Raman tensors of Raman-active $A_g^{(1)}$ modes in $ZrTe_5$ is given by

$$R[A_g^{(1)}] = \begin{bmatrix} a & 0 & 0 \\ 0 & a & 0 \\ 0 & 0 & c \end{bmatrix} = \begin{bmatrix} |a|e^{i\varphi_a} & 0 & 0 \\ 0 & |a|e^{i\varphi_a} & 0 \\ 0 & 0 & |c|e^{i\varphi_c} \end{bmatrix} \tag{S1}$$

With light polarization within the a-c-plane, its Raman intensity can be expressed as

$$I(A_g^{(1)}) \propto |a|^2 \sin^4\theta + |c|^2 \cos^4\theta + 2|a||c|\cos(\psi_c - \psi_a)\sin^2\theta\cos^2\theta, \tag{S2}$$

where θ is defined as the angle between the c-axis and incident light polarization. And with $\frac{\partial \epsilon_a}{\partial Q} = |a|e^{i\psi_a}$, we have[?]

$$\frac{I(A_g^{(1)})_a}{I(A_g^{(1)})_c} = \frac{|a|^2}{|c|^2} = \frac{\left|\frac{\partial \epsilon_a}{\partial Q}\right|^2}{\left|\frac{\partial \epsilon_c}{\partial Q}\right|^2} \tag{S3}$$

Thus, we moved each atom based on the atomic displacement vector of the $A_g^{(1)}$ vibrational mode and used VASP to calculate the dielectric constant spectrum for different atomic displacement amounts Q. In our experiment, we computed $\frac{\partial \epsilon_a}{\partial Q}$ and $\frac{\partial \epsilon_c}{\partial Q}$ at 1.9 eV (650 nm). The calculation results and experimental results are both shown in Figure. S1. The small deviations arise from differences in lattice parameters between the real and calculated values. Their good agreement certificate the reliability of first-principles calculation results.

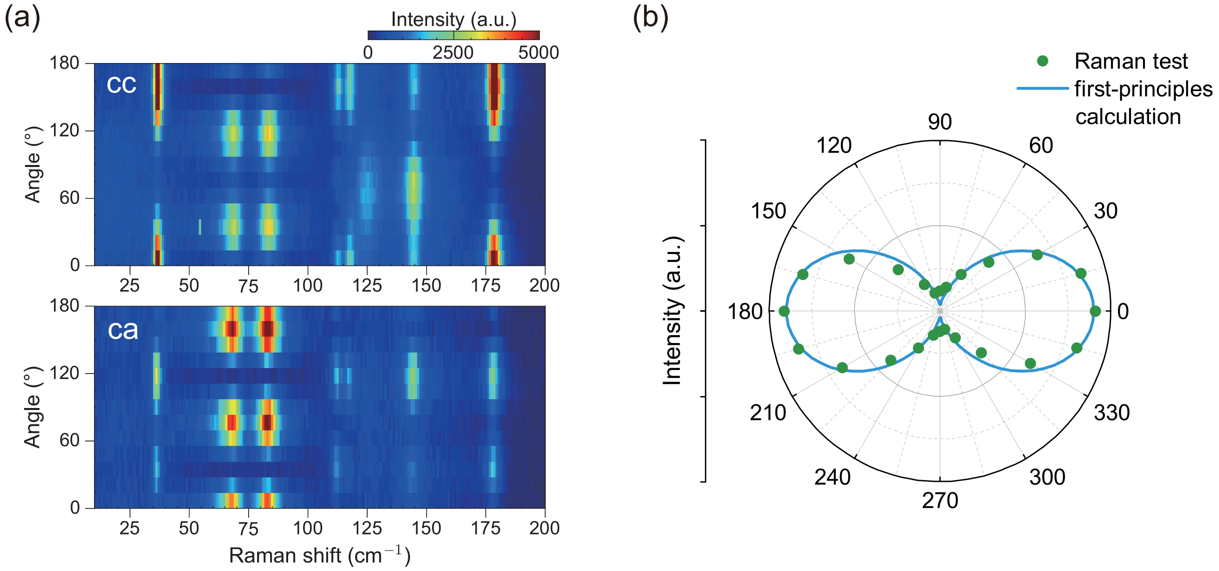

Figure S1: The experimental and calculation results of probe-polarization-dependent Raman intensity. (a) The upper figure is measured with the polarization of incident laser and analyzer both parallel to c-axis (cc). The lower figure is measured with ca polarization setup. The angle between crystal c-axis and $\theta = 0°$ is 13.11° fitted by the result. (b) The dependence of Raman intensity on probe polarization. (The experimental result was rotated 13.11°)

2 Results of real-time time-independent density functional theory (rt-TDDFT) simulations.

To characterize the displacive excitation of coherent phonons, we performed theoretical stimulations. We first implemented density functional theory (DFT) calculations in order to derive the structure of bulk $ZrTe_5$ equilibrium and eigenvectors of phonons. Subsequently, we implemented real-time time-independent density functional theory (rt-TDDFT) simulations, investigating the displacive excitation amplitudes of A_g mode by a femtosecond laser pulse.

2.1 Calculational Details

We performed our DFT calculations by the Vienna ab initio simulation package (VASP)[1] and used the Phonopy software package[2] for phonon calculations. We applied pseudopotentials generated by the Projected Augmented Wave (PAW) method[3] and the exchange-correlation function in Perdew-Burke-Ernzerhof (PBE) of generalized gradient approximation (GGA)[4]. The van der Waals (vdW) corrected optB86b-vdw functional[5] was taken into account throughout our DFT calculations, and a Γ-centered $7 \times 7 \times 4$ k-point-mesh sampling of the Brillouin zone was used for primitive cell.

Our rt-TDDFT simulations were implemented by the PWmat package.[6,7] The rt-TDDFT calculations also relied on PBE exchange-correlation function but with DFT-D3[8] vdW correction. A $7 \times 2 \times 2$ k-point-mesh sampling generated by Monkhorst-Pack method[9] was applied for $\sqrt{2} \times \sqrt{2} \times 1$ cell. The step length is 0.05 fs.

2.2 Results

Equilibrium structure is required before investigating the displacive excitation of bulk $ZrTe_5$. Hence, we first performed structure relaxation. The space group of bulk $ZrTe_5$ is Cmcm, in which two Zr atoms as well as two Te atoms occupy 4c-Wyckoff positions, and eight Te atoms occupy 8f positions. Our calculation results suggest the lattice constants are a = 3.99 Å, b = 14.53 Å and c = 13.72 Å, which is in agreement with experimentally observed structure. Then the phonon spectrum of the optimized structure was calculated, and frequencies of relevant A_g modes at Γ point are listed in Table. S1, which is consistent with experimental data in the main text.

Table S1: The calculated zone center optical phonon frequencies for A_g symmetry modes of bulk $ZrTe_5$.

**Mode label**	**Frequency in THz (cm^{-1})**
$A_g^{(1)}$	1.159 (38.67)
$A_g^{(2)}$	3.355 (111.96)
$A_g^{(3)}$	3.435 (114.64)
$A_g^{(4)}$	4.342 (144.90)
$A_g^{(5)}$	5.024 (167.68)
$A_g^{(6)}$	6.849 (228.56)

Based on the computed phonon spectrum, we can perform rt-TDDFT simulation to investigate the atomic placement and phonon mode amplitude induced by a femtosecond pump. A Gaussian laser pump with a peak electric field of 0.118 eV/Å and full width at half maximum (FWHM) of 160 fs applied propagates along the b-axis with linear polarization direction of c-axis. The phonon mode amplitude is acquired by projecting atomic displacement on eigenvectors of phonons, as depicted in Figure. S2. It's apparent that $A_g^{(1)}$ mode is successfully excited, and exceeds other A_g modes in atomic displacement Q.

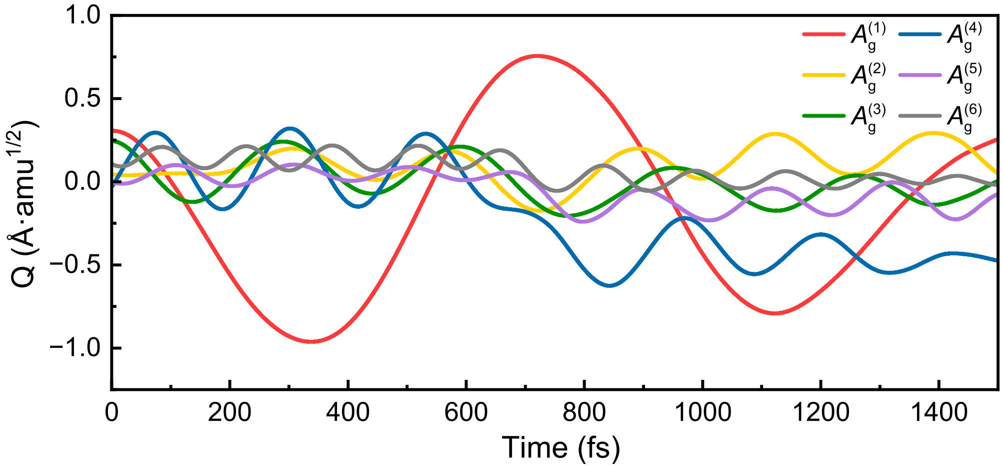

Figure S2: The time-dependent amplitude Q of mode $A_{\mathrm{g}}^{(1)}$ excited by Gaussian laser pump shown in the panel.

After Fourier transformation of Q, the amplitude of 0-THz item is 0.268 $\text{Å} \cdot \text{amu}^{1/2}$ which represents the shift of phonon equilibrium position, and the amplitude of 1.16-THz item is 0.687 $\text{Å} \cdot \text{amu}^{1/2}$ which represents the amplitude of phonon vibration.

3 The necessity of bi-exponential fitting for low temperature range.

In the following Figure. S4, we provide part of the single-exponential fitting results for the entire temperature range. We can see that as temperature decreasing, the contribution of a long-lived process is becoming increasingly important. Below approximately 150 K, a single-exponential fitting model is no longer adequate, necessitating an additional exponential term for better fitting. Thus, we employ the following bi-exponential equation Eq. S4 to fit the experimental data below 150 K, the fitting results are shown in Figure. S3, we can get good agreements at all temperatures.

$$\frac{\Delta R(t)}{R} = \left(A_{\mathrm{ep}} \exp\left(-\frac{t-t_0}{\tau_{\mathrm{ep}}}\right) + A_{\mathrm{e2}} \exp\left(-\frac{t-t_0}{\tau_{\mathrm{e2}}}\right) + A_{\mathrm{op}} \exp\left(-\frac{t-t_0}{\tau_{\mathrm{op}}}\right) \sin(\omega t + \phi) + C \right) \otimes G(t). \tag{S4}$$

Here, A_{ep} and τ_{ep} represents the amplitude and relaxation time of the short-lived term, while A_{e2} and τ_{e2} stands for the long-lived term.

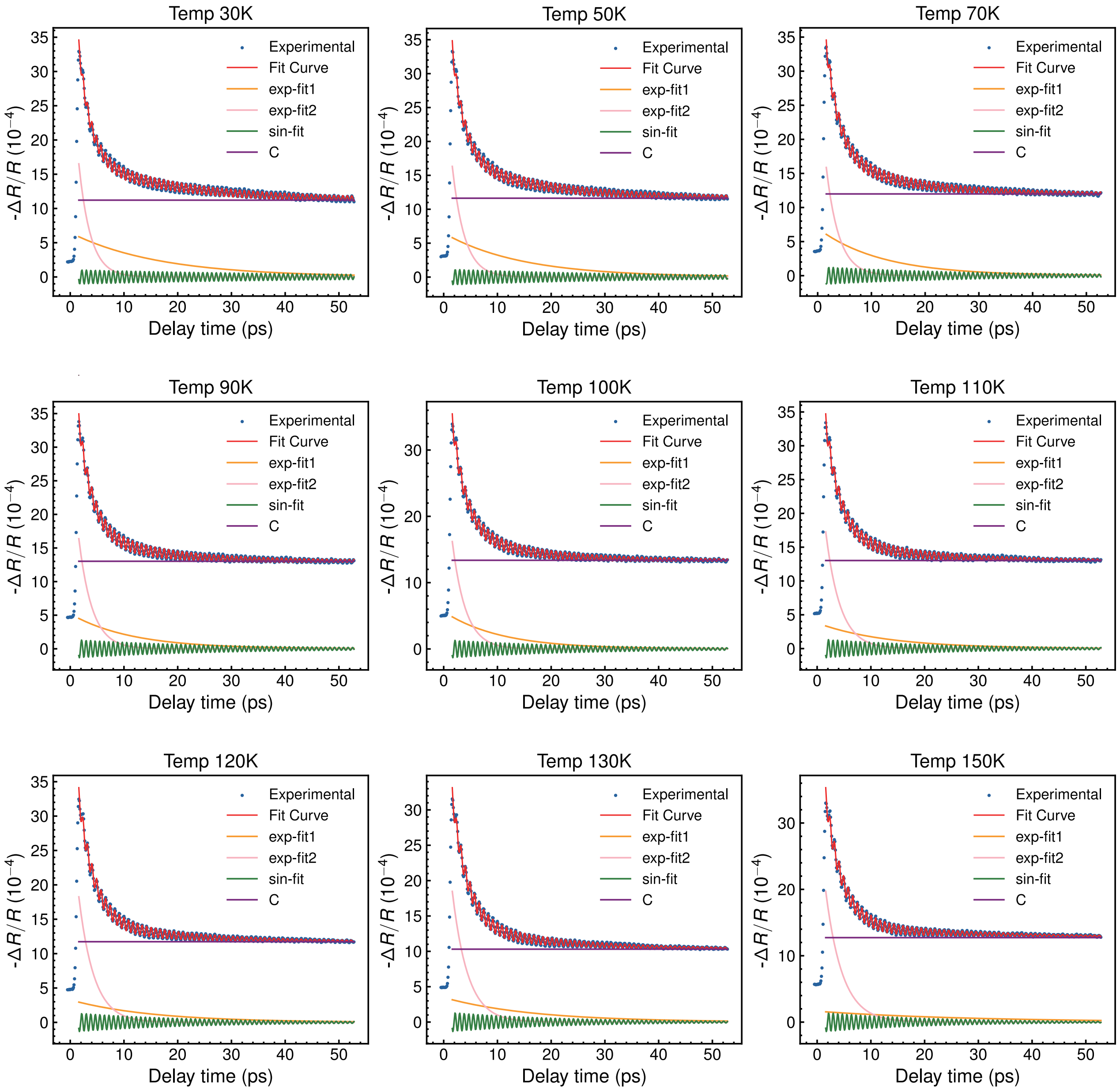

Figure S3: The fitting result of bi-exponential decay.

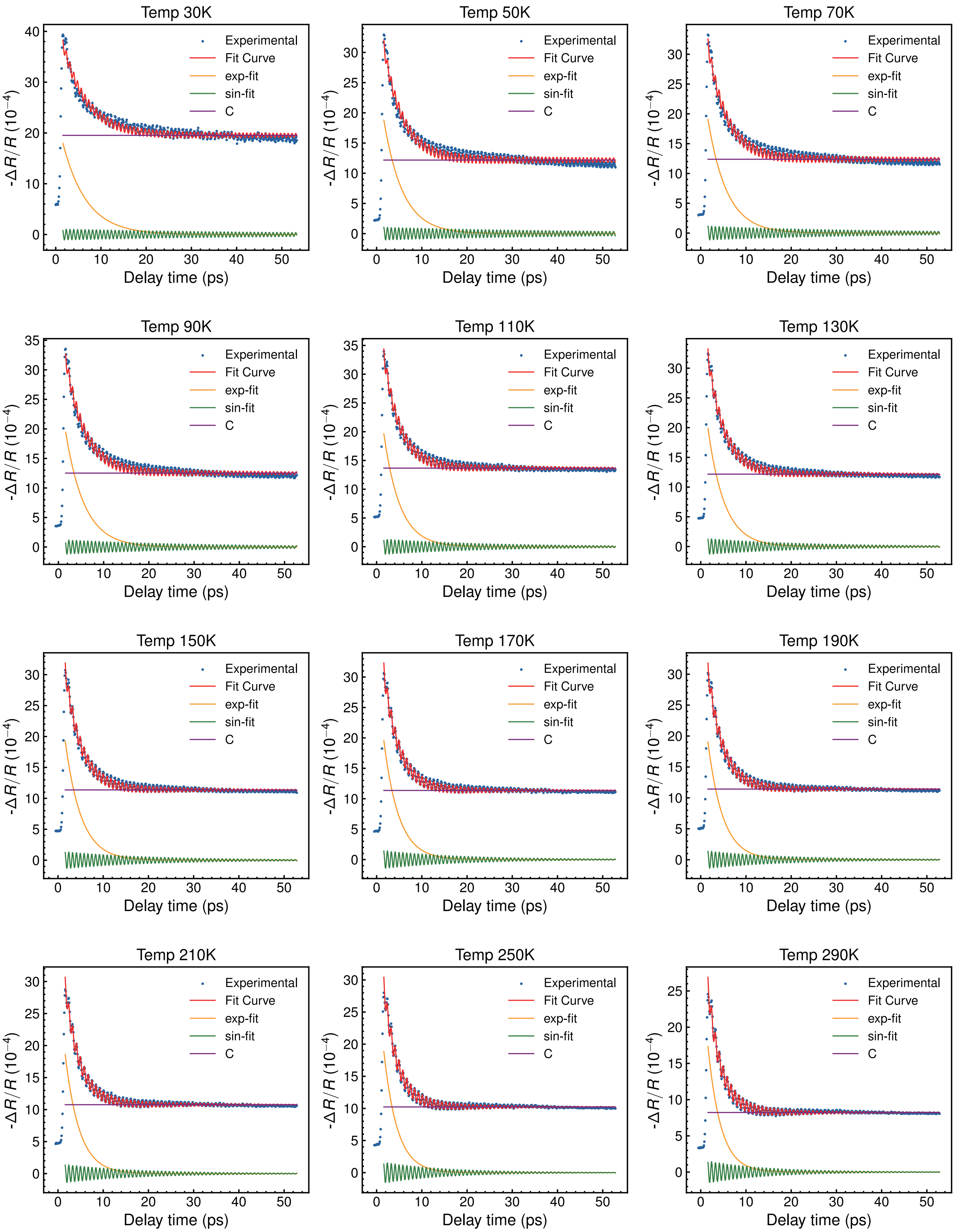

Figure S4: The fitting result of single-exponential decay.

The amplitude of the long-lived term A_{e2} decreases when temperature is increasing (shown in Figure. S5), which may suggest a connection to significant topological surface state signals at low temperatures.

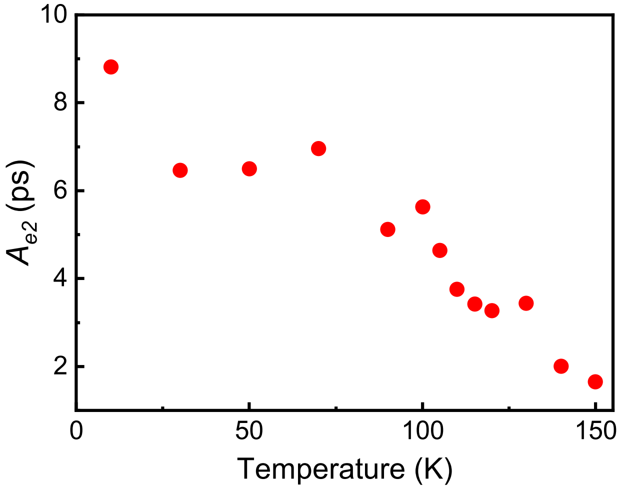

Figure S5: A_{e2} as a function of temperature.

4 The limitations of the Two-Temperature Model (TTM) at low temperatures and its applicable temperature range in this work.

In the TTM, we treat both the electrons and lattice as classical systems for discussion, with each maintained in a thermalized state. The TTM describes the flow of heat between electrons and lattice when $T_e > T_l$ after laser excitation. We define the electron-phonon relaxation time τ_{ep}, the energy transfer rate $H(T_e, T_l)$, and $f(T)$ by Eq. 12-14 in the main text. In $f(T)$, we introduced the Debye temperature, and it determines the upper limit of the integral in energy transfer rate H.

According to the definition of Debye temperature $\Theta_D = \frac{\hbar}{k_B}\left(\frac{6\pi^2 n}{V}\right)^{1/3} v_s$, for $ZrTe_5$ we have $\Theta_D \approx 165\,K$ $(\rho = 5.44 g/cm^3, v_s = 3440 m/s)$. It is consistent with the Debye temperature of $ZrTe_5$ (154 K) derived from both temperature-dependent heat capacity and DFT-derived phonon group velocities by Jie Zhu et al[10]. According to the equations above, for $T_l \ll \Theta_D$, the function $g(T_l)$ varies as T_l^4, and $\tau_{ep} \propto T_l^{-3}$. Their temperature-dependent variations are illustrated in Figure S6. However, the experimental data of nearly all the temperature-dependent τ_{ep} do not show an increase as temperature decreases. This is the limitation of TTM at low temperatures.

One of the fundamental assumptions of TTM is that both the electrons and lattice are in a thermalized state. However, Rogier H. M. Groeneveld et al. demonstrated in their study of electron-electron and electron-phonon relaxation in Au and Ag[11] that, at very low temperatures, the electron-electron relaxation time is longer than the typical electron-phonon relaxation time. This indicates that the electron system has not reached a thermalized state before exchanging heat with the lattice. Therefore, they proposed a Nonthermal Electron Model (NEM), which considers a nonthermal electron gas coupled to the phonon system. Therefore, we mentioned in the main text that the deviation between the experimental data and TTM predictions at low temperatures in Figure 5 arises from the neglection of the significant contribution from e-e scattering at low temperatures. Referring to the NEM, we calculated the thermalization time of the electron system τ_{th}. We found that when $\tau_{th} = \tau_{ep}$, the temperature of the thermalized distribution is below 260 K, and the initial temperature (ambient temperature) should be

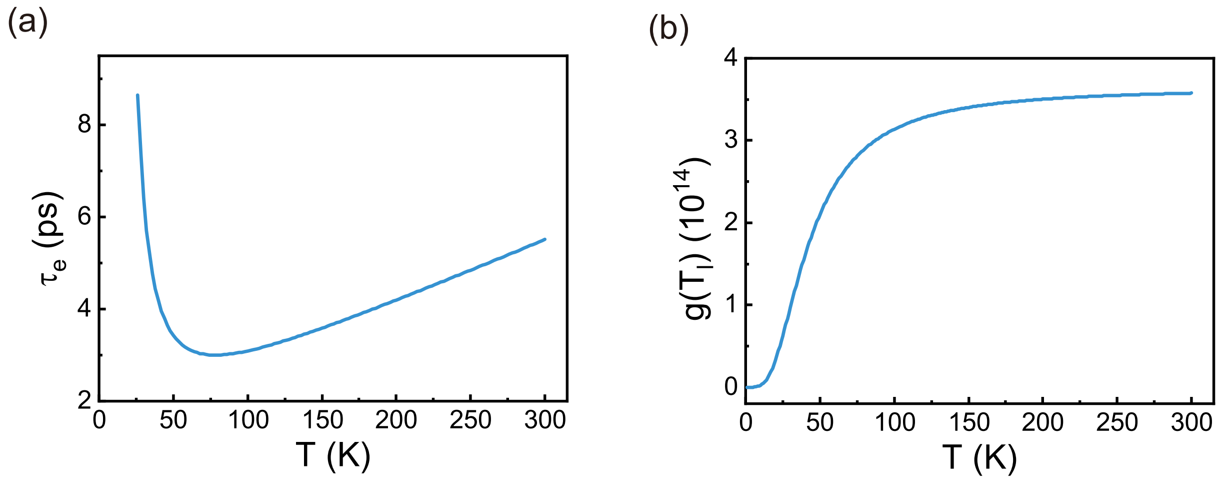

Figure S6: The temperature dependence of $g(T_l)$ and τ_e.

below 70 K. Since electron scattering in $ZrTe_5$ is stronger than in Ag and Au, the temperature at which $\tau_{th} = \tau_{ep}$ will also be lower. We believe that this temperature determines the limitations of the TTM at low temperatures. Previous studies[12,13] suggested that this temperature is approximately $\frac{1}{5}\Theta_D$. Our experimental results significantly deviate from the TTM predictions at T >150 K, thus the temperature range applicable to the TTM in $ZrTe_5$ is 50-150 K.

5 Analysis of characteristic parameters for the short-lived process as a function of pump fluence.

To further confirm our assumption that the inflection point in electron relaxation time is caused by Lifshitz transition, we conducted pump-fluence-dependent experiments at different temperatures, and obtained the relaxation time (τ_{ep}) and amplitude (A_{ep}) of the short-lived process as a function of pump fluence.

At temperatures below 105 K (taking 70 K as an example), Fermi energy is in the valence band of $ZrTe_5$ according to previous research[14]. The relationship between A_{ep} and τ_{ep} with pump fluence (F) is shown by red dots in Figure. S7. We use TTM to fit these data as mentioned in the main text.

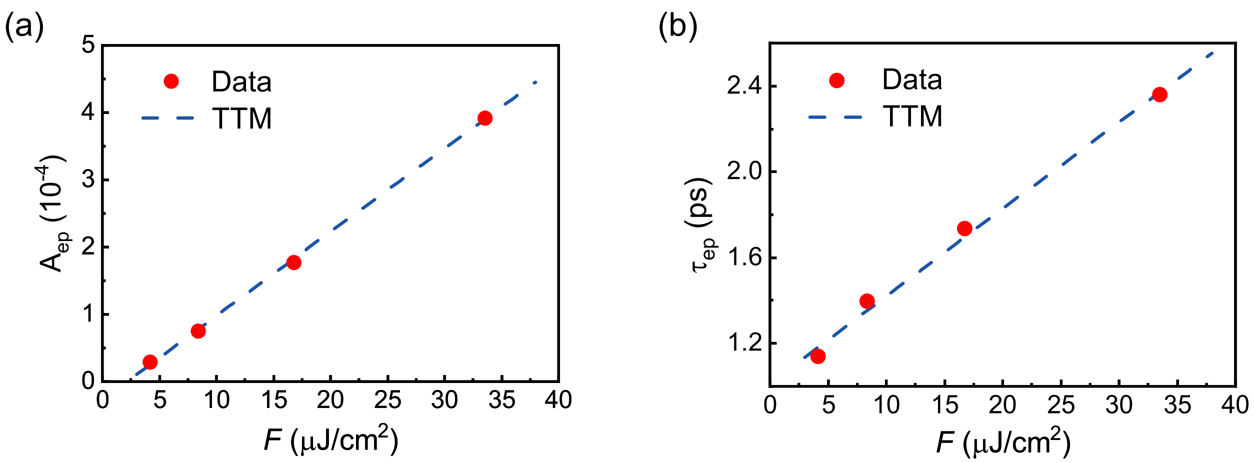

Figure S7: Experimental results and TTM simulation of A_{ep} and τ_{ep} at 70 K.

As verified by previous experiments, the exponential decay term represents the absorption of light by

excited state electrons. Thus, its amplitude A_{ep} is given by the difference in the densities of electrons before and after photoexcitation. In a Landau Fermi liquid, the density of electrons is proportional to the electronic temperature $n_{T_e} \propto T_e$. And under the assumption that after photoexcitation all the absorbed energy initially goes into the electronic system, we have

$$A_{\mathrm{ep}} \propto n_p \;=\; n_{T_e} - \; n_{T_0} \propto T_e - T_0. \tag{S5}$$

Here, T_0 is the initial temperature of the electron system, which is equivalent to room temperature. T_e is the elevated temperature of the electron system after thermalization. Therefore, the $A_{\mathrm{ep}} - F$ curve, fitted with T_e calculated from the TTM, is represented by the blue dashed line in Figure. S7a. It increases with increasing pump fluence. Using g_∞ obtained from the fitting in the main text, we performed numerical calculations on Eq. S6 for the general expression of TTM.

$$C_e(T_e)\frac{\partial T_e}{\partial t} = \frac{\partial}{\partial z}\left(\kappa_e(T_e)\frac{\partial T_e}{\partial z}\right) - g(T_e - T_l) + S(z,t), C_l\frac{\partial T_l}{\partial t} = g(T_e - T_l). \tag{S6}$$

Electron thermal conductivity $\kappa_e \sim 5 \times 10^{-3}\mathrm{W} \cdot \mathrm{m}^{-1} \cdot \mathrm{K}^{-1}$ according to previous work[15]. The electron temperature increases to $\sim 300\,\mathrm{K}$ after absorbing the pump energy in our experiment (shown in Figure S8a), which does not satisfy the condition $T_e - T_l \ll T_l$. Therefore, τ_{ep} should be expressed as

$$\tau_{\mathrm{ep}} = \frac{\gamma(T_e + T_l)}{2g(T_l)}. \tag{S7}$$

during our calculation and fitting.

By combining Eq. 11 with the numerical calculation results, we obtained the corrected $\gamma = 0.67\,\mathrm{mJ} \cdot \mathrm{mol}^{-1}{\cdot}\mathrm{K}^{-2} = 5\,\mathrm{J}{\cdot}\mathrm{m}^{-3}{\cdot}\mathrm{K}^{-2}$ using Eq. S7, and calculated electron-phonon coupling factor $\lambda\langle\omega^2\rangle = \frac{g_{ep}\pi k_B}{3\gamma\hbar} \simeq 9.49 \times 10^{25}\,\mathrm{Hz}^2 \simeq 4.11\,\mathrm{meV}^2$ [16,17]. Furthermore, we obtained $\tau_{\mathrm{ep}} - F$ using numerical calculation of T_e under the condition of $T_e - T_l \sim T_l$, as shown by the blue dashed line in Figure. S7b. Simulation results are in good agreement with experimental results.

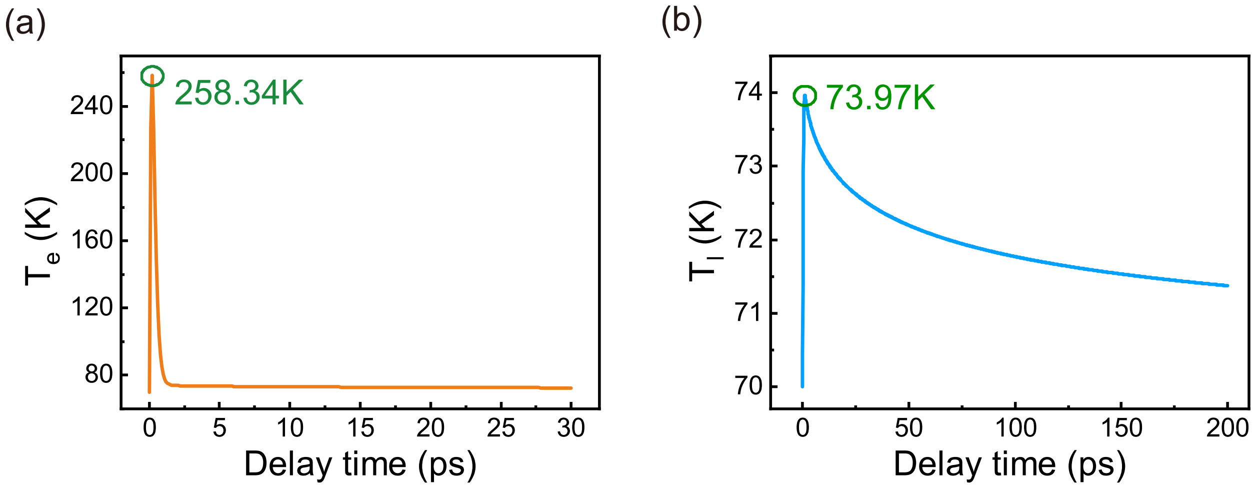

Figure S8: (a) The time evolution of the electron temperature T_e, $T_e(max) \;= 258.34K$. (b) The time evolution of the lattice temperature T_l, $T_l(max) \;= 73.97K$.

In temperature-dependent experiments, the thermometer-measured temperature is used to represent the lattice temperature. To validate the reliability of this approach, we employed the Finite Difference Method to compute the time evolution of the electron temperature T_e and the lattice temperature T_l under experimental conditions using the TTM equations Eq. S6. The results at 70 K shown in

Figure S8b indicate that the maximum lattice temperature $T_l(max)$ is approximately equal to the ambient temperature, with the steady-state lattice temperature at the focal point of the laser beam increasing by less than 2%.

At temperatures above 195 K (taking 230 K as an example), Fermi energy is in the valence band of $ZrTe_5$. When the excited electrons relax to the Fermi energy, they have to cross a small band gap, A_{ep} and τ_{ep} as a function of F are shown by red dots in Figure. S9.

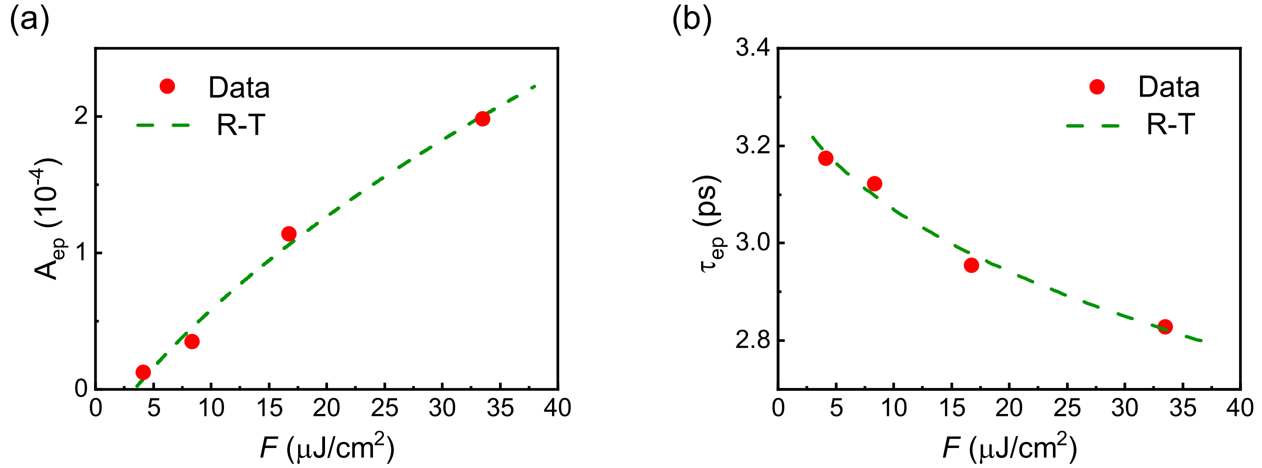

Figure S9: Experimental results and RT simulation of A_{ep} and τ_{ep} at 230 K.

Accoridng to the discussion in the main text, n_s in RT model is related to the initial concentrations of electron-hole pairs n_0 and high frequency phonons N_0. In previous work[18], the saturation pump fluence of density of state in the electronic energy band is $\sim 50\,\mu J/\text{cm}^2$, similar trend was also observed in our pump fluence-dependent experiments at $300\,K$ as shown in Figure. S10a.

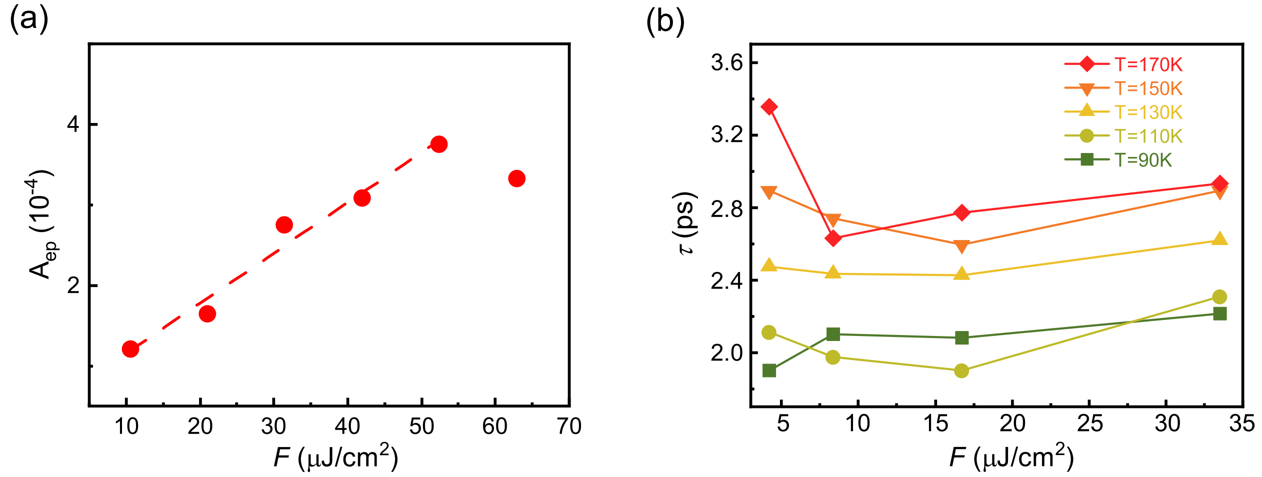

Figure S10: (a) A_{ep} as a function of F at 300 K. (b) τ_{ep} as a function of F at 105 K to 195 K.

Thus, in the relatively low pump fluence regime($< 33\,\mu J/\text{cm}^2$), we have n_0 and N_0 propotional to F, and $n_s \propto \sqrt{1+cF} - 1$. Considering the temperature, the number of thermally excited carriers is strongly suppressed[12], and we have $A_{\text{ep}} \propto n_s$. The $A_{\text{ep}} - F$ data fitted by $A_{\text{ep}} \propto \sqrt{1+cF-1}$ is shown by green dashed line in Figure. S9a. It increases as F increasing.

According to Eq. 13 in the main text, the fluence dependence of τ_{ep} is also governed by the fluence dependence of n_s. Thus, we fit $\tau_{\text{ep}} - F$ data by $\tau_{\text{ep}}^{-1} \propto \sqrt{1+cF-1}$, and the result is shown in Figure. S9b. However, it decreases with increasing pump fluence, showing an opposite trend to that of TTM.

For temperatures above 105 K and below 195 K, τ_{ep} does not change monotonically with F as shown in Figure. S10b. Based on the above conclusions and previous works by ARPES, we attribute this to the Fermi energy locating in the bandgap of $ZrTe_5$ within this temperature range. Only when the temperature rise caused by the pump fluence is large enough to excite some of the electrons to the conduction band, will the contribution of TTM process gradually become significant. It is reflected by τ_{ep} increase at large pump fluence in $\tau_{ep} - F$ experiments.

6 Discussion of the potential mechanism of the long-lived process.

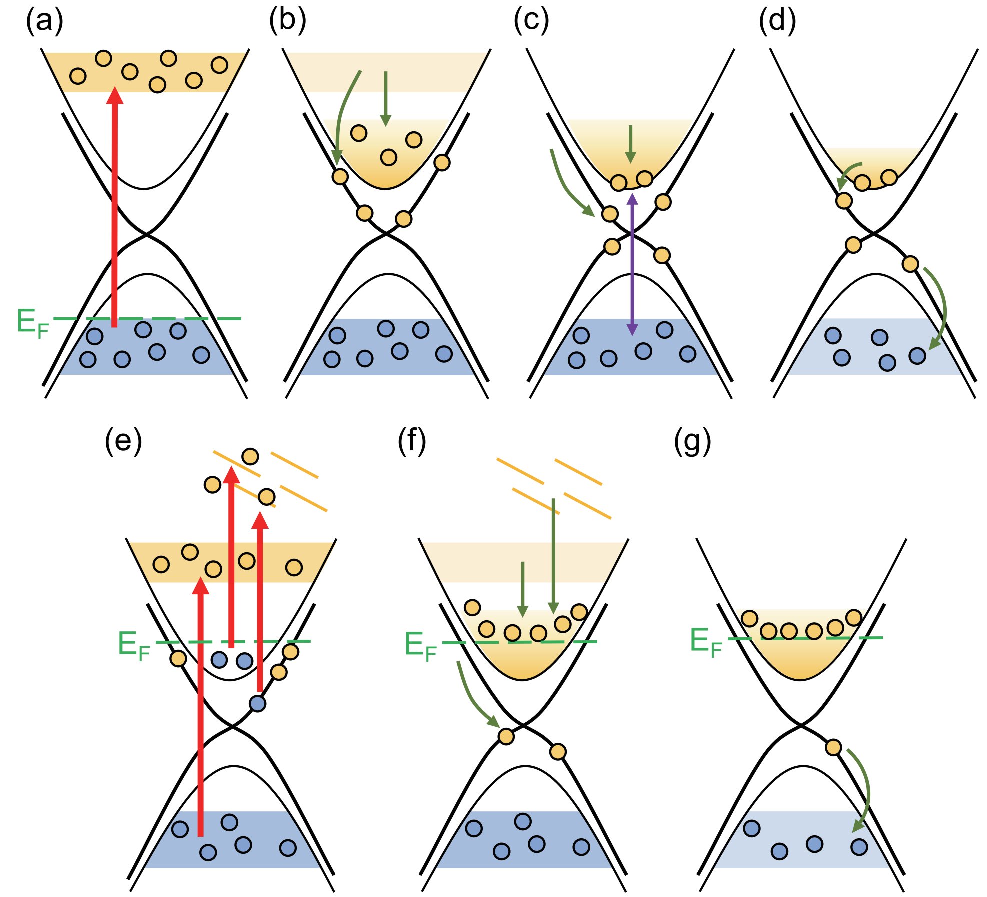

Figure S11: (a-d) The relaxation mechanism of TSS at temperatures above 100 K. (a) The direct transition induced by the pump laser. (b) Scattering into topological surface state (TSS) and bulk conduction band (BCB). (c) Intraband scattering of the BCB and TSS. (d) The recombination of TSS electrons and bulk valence band (BVB) holes. (e-g) The relaxation mechanism of TSS at temperatures below 100 K.

Based on previous ARPES experiments of weak topological insulator $ZrTe_5$,[19] the electronic band structure of the a-b surface exhibits a surface state with a linear dispersive Dirac cone structure. Thus, the relaxation of excited electrons is depicted in the following Figure S11. Figure S11(a-d) illustrate the relaxation mechanism when the Fermi energy lies within the valence band (T>100 K). Upon irradiation by the pump pulse, electrons near the Fermi level absorb photon energy and are excited to the conduction band, simultaneously leaving behind holes in the valence band. Subsequently, the excited electrons undergo relaxation either to the conduction band minimum or to the surface states, as illustrated by the green arrows in Figure S11b. The recombination of electrons at the conduction band minimum with holes in the valence band, indicated by the purple arrow in Figure S11c, corresponds to the

short-lived relaxation process (<3 ps). In contrast, the relaxation of surface state electrons exhibits a longer timescale, the origin of which will be discussed in detail later. As surface state electrons recombine with valence band holes, additional electrons from the conduction band minimum continue to populate the surface states, further extending its relaxation time. Figure S11(e-g) illustrate the relaxation mechanism when the Fermi energy lies within the conduction band (T<100 K). Upon pump pulse excitation, electrons in the valence band, conduction band minimum, and surface states absorb photon energy and are excited to higher energy levels. Subsequently, these excited electrons relax toward the Fermi level, corresponding to the short-lived relaxation. Meanwhile, surface state electrons near the Fermi level relax into the valence band and recombine with holes, giving rise to the long-lived relaxation component.

Previous time-resolved studies on the surface states of classical topological insulators such as Bi_2Se_3,[20,21] Bi_2Te_3,[22] and Bi_2Te_2Se[23] have shown that the electron relaxation time of the surface states exceeds 10 ps. This is approximately an order of magnitude longer than the relaxation time observed for bulk band electrons. Based on these findings, we propose that the long-lived relaxation process of the topological surface states in $ZrTe_5$ may be attributed to two key factors: 1) Low backscattering: The surface states of topological insulators are protected by time-reversal symmetry, exhibiting spin-momentum locking. This characteristic prohibits elastic backscattering of surface state electrons, significantly reducing scattering events. Therefore, this results in longer relaxation times for surface state electrons as shown in Figure S11c. 2) Bulk-surface interaction: As illustrated in Figure S11d, bulk-to-surface scattering continuously populates the topological surface states (TSS), resulting in a longer lifetime. Excitation with any above-band-gap photon energy should result in the same TSS filling behavior, as both inter- and intraband scattering processes inevitably lead to carrier relaxation toward the bulk conduction band (BCB) edge.[24] This supports the possibility of bulk-to-surface scattering occurring in our experiment.

To strengthen our interpretation, we provide an extended analysis of the long-lived component's temperature dependence with temperature-dependent TSS relaxation time τ_L shown in Figure S12.

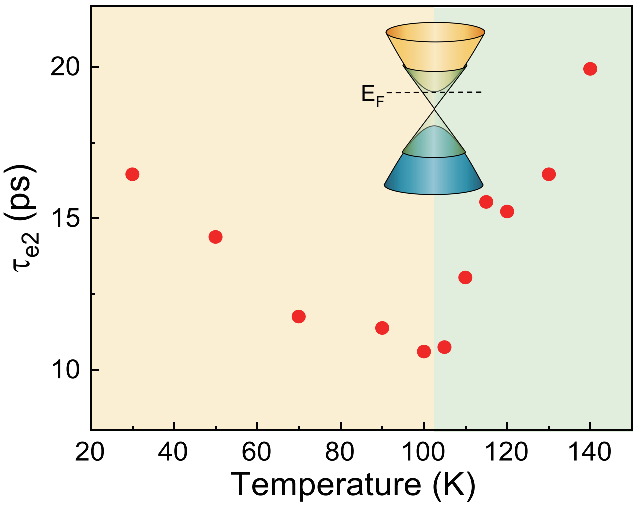

Figure S12: Temperature-dependent TSS relaxation time τ_{e2}.

Based on the mechanism mentioned above, we can infer that: Below 100 K, the Fermi level resides within the conduction band, enabling surface state electrons to relax to the valence band immediately following laser excitation. Consequently, temperature influences only the coupling between surface state electrons and phonons, without modifying the overall relaxation process. Thus, as the temperature rises, the electron-phonon coupling becomes stronger, leading to a reduction in relaxation time. Above

100 K, the Fermi level is situated within the surface state. Additional scattering from bulk conduction band (BCB) to topological surface states (TSS) introduces a T-dependent τ_L. Consequently, as the temperature rises, the Fermi level decreases, leading to an increase in electron lifetime. The monotonic increase in the amplitude of the long-lived process as the temperature decreases can be attributed to the reduced thermal excitation of bulk state electrons at low temperatures, which makes the effects of surface states more significant.

7 Experimental setup of ultrafast pump-probe system.

The experimental set-up of the ultrafast pump-probe system in this work.

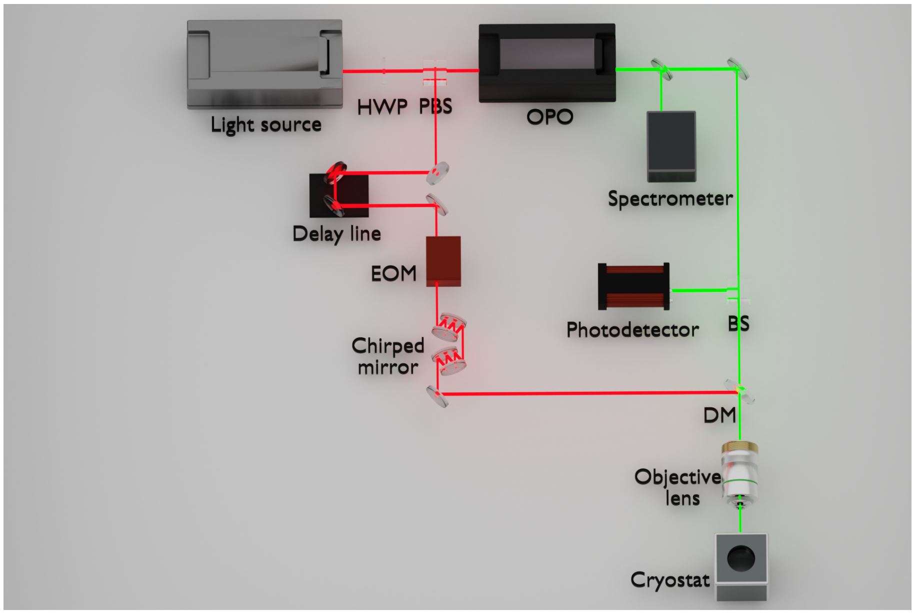

Figure S13: Schematic layout for ultrafast pump-probe system.

References

[1] G. Kresse, J. Furthmüller, Efficient iterative schemes for ab initio total-energy calculations using a plane-wave basis set, *Physical review B* **1996**, *54*, 16 11169.

[2] A. Togo, L. Chaput, T. Tadano, I. Tanaka, Implementation strategies in phonopy and phono3py, *Journal of Physics: Condensed Matter* **2023**, *35*, 35 353001.

[3] P. E. Blöchl, Projector augmented-wave method, *Physical review B* **1994**, *50*, 24 17953.

[4] J. P. Perdew, K. Burke, M. Ernzerhof, Generalized gradient approximation made simple, *Physical review letters* **1996**, *77*, 18 3865.

[5] J. Klimeš, D. R. Bowler, A. Michaelides, Van der Waals density functionals applied to solids, *Physical Review B—Condensed Matter and Materials Physics* **2011**, *83*, 19 195131.

[6] B. Kanungo, V. Gavini, Real time time-dependent density functional theory using higher order finite-element methods, *Physical Review B* **2019**, *100*, 11 115148.

[7] W.-H. Liu, Z. Wang, Z.-H. Chen, J.-W. Luo, S.-S. Li, L.-W. Wang, Algorithm advances and applications of time-dependent first-principles simulations for ultrafast dynamics, *Wiley Interdisciplinary Reviews: Computational Molecular Science* **2022**, *12*, 3 e1577.

[8] S. Grimme, J. Antony, S. Ehrlich, H. Krieg, A consistent and accurate ab initio parametrization of density functional dispersion correction (DFT-D) for the 94 elements H-Pu, *The Journal of chemical physics* **2010**, *132*, 15.

[9] H. J. Monkhorst, J. D. Pack, Special points for Brillouin-zone integrations, *Physical review B* **1976**, *13*, 12 5188.

[10] J. Zhu, T. Feng, S. Mills, P. Wang, X. Wu, L. Zhang, S. T. Pantelides, X. Du, X. Wang, Record-low and anisotropic thermal conductivity of a quasi-one-dimensional bulk ZrTe5 single crystal, *ACS applied materials & interfaces* **2018**, *10*, 47 40740.

[11] R. H. Groeneveld, R. Sprik, A. Lagendijk, Femtosecond spectroscopy of electron-electron and electron-phonon energy relaxation in Ag and Au, *Physical Review B* **1995**, *51*, 17 11433.

[12] J. Demsar, J. L. Sarrao, A. J. Taylor, Dynamics of photoexcited quasiparticles in heavy electron compounds, *Journal of Physics: Condensed Matter* **2006**, *18*, 16 R281.

[13] K. Ahn, M. Graf, S. Trugman, J. Demsar, R. Averitt, J. Sarrao, A. Taylor, Ultrafast quasiparticle relaxation dynamics in normal metals and heavy-fermion materials, *Physical Review B* **2004**, *69*, 4 045114.

[14] Y. Zhang, C. Wang, L. Yu, G. Liu, A. Liang, J. Huang, S. Nie, X. Sun, Y. Zhang, B. Shen, et al., Electronic evidence of temperature-induced Lifshitz transition and topological nature in ZrTe5, *Nature communications* **2017**, *8*, 1 15512.

[15] M. Hooda, C. Yadav, Enhanced thermopower and low thermal conductivity in p-type polycrystalline ZrTe5, *Applied Physics Letters* **2017**, *111*, 5.

[16] P. B. Allen, Theory of thermal relaxation of electrons in metals, *Physical review letters* **1987**, *59*, 13 1460.

[17] V. E. Gusev, O. B. Wright, Ultrafast nonequilibrium dynamics of electrons in metals, *Physical Review B* **1998**, *57*, 5 2878.

[18] N. Li, W. Liang, S.-N. Luo, Hot carrier dynamics and phonon anharmonicity of ZrTe5 revealed with femtosecond transient optical spectroscopy, *Physical Review B* **2020**, *101*, 1 014304.

[19] P. Zhang, R. Noguchi, K. Kuroda, C. Lin, K. Kawaguchi, K. Yaji, A. Harasawa, M. Lippmaa, S. Nie, H. Weng, et al., Observation and control of the weak topological insulator state in ZrTe5, *Nature communications* **2021**, *12*, 1 406.

[20] S. Sim, M. Brahlek, N. Koirala, S. Cha, S. Oh, H. Choi, Ultrafast terahertz dynamics of hot Dirac-electron surface scattering in the topological insulator Bi2Se3, *Physical Review B* **2014**, *89*, 16 165137.

[21] A. Crepaldi, F. Cilento, B. Ressel, C. Cacho, J. Johannsen, M. Zacchigna, H. Berger, P. Bugnon, C. Grazioli, I. Turcu, et al., Evidence of reduced surface electron-phonon scattering in the conduction band of Bi2Se3 by nonequilibrium ARPES, *Physical Review B—Condensed Matter and Materials Physics* **2013**, *88*, 12 121404.

[22] M. Hajlaoui, E. Papalazarou, J. Mauchain, G. Lantz, N. Moisan, D. Boschetto, Z. Jiang, I. Miotkowski, Y. Chen, A. Taleb-Ibrahimi, et al., Ultrafast surface carrier dynamics in the topological insulator Bi2Te3, *Nano letters* **2012**, *12*, 7 3532.

[23] V. Iyer, Y. P. Chen, X. Xu, Ultrafast surface state spin-carrier dynamics in the topological insulator Bi2Te2Se, *Physical review letters* **2018**, *121*, 2 026807.

[24] J. A. Sobota, S. Yang, J. G. Analytis, Y. Chen, I. R. Fisher, P. S. Kirchmann, Z.-X. Shen, Ultrafast Optical Excitation of a Persistent Surface-State Population in the Topological Insulator Bi2Se3, *Physical review letters* **2012**, *108*, 11 117403.
